# Establishment of an ovarian cancer exhausted CD8+T cells-related genes model by integrated analysis of scRNA-seq and bulk RNA-seq

**DOI:** 10.1186/s40001-024-01948-8

**Published:** 2024-07-05

**Authors:** Tian Hua, Deng-xiang Liu, Xiao-chong Zhang, Shao-teng Li, Jian-lei Wu, Qun Zhao, Shu-bo Chen

**Affiliations:** 1 Department of Gynecology, Affiliated Xingtai People Hospital of Hebei Medical University, Xingtai, China; 2 Department of Oncology, Affiliated Xingtai People Hospital of Hebei Medical University, 16 Hongxing Road, Xingtai, Hebei 054001 People’s Republic of China; 3 Department of Gynecological Oncology, Shandong Cancer Hospital and Institute, Shandong First Medical University and Shandong Academy of Medical Sciences, 440 Jiyan Road, Jinan, Shandong 250021 People’s Republic of China; 4 The Third Department of Surgery , Hebei Medical University, Fourth Hospital, Road Jiankang No. 12, Hebei, 050001 People’s Republic of China; 5 Hebei Key Laboratory of Precision Diagnosis and Comprehensive Treatment of Gastric Cancer, Shijiazhuang, China

**Keywords:** Ovarian cancer, Exhausted CD8+T cells, Prognostic signature, Single-cell RNA-sequencing, Homologous recombination repair deficiency

## Abstract

Ovarian cancer (OC) was the fifth leading cause of cancer death and the deadliest gynecological cancer in women. This was largely attributed to its late diagnosis, high therapeutic resistance, and a dearth of effective treatments. Clinical and preclinical studies have revealed that tumor-infiltrating CD8+T cells often lost their effector function, the dysfunctional state of CD8+T cells was known as exhaustion. Our objective was to identify genes associated with exhausted CD8+T cells (CD8TEXGs) and their prognostic significance in OC. We downloaded the RNA-seq and clinical data from the Cancer Genome Atlas (TCGA) and Gene Expression Omnibus (GEO) databases. CD8TEXGs were initially identified from single-cell RNA-seq (scRNA-seq) datasets, then univariate Cox regression, the least absolute shrinkage and selection operator (LASSO), and multivariate Cox regression were utilized to calculate risk score and to develop the CD8TEXGs risk signature. Kaplan–Meier analysis, univariate Cox regression, multivariate Cox regression, time-dependent receiver operating characteristics (ROC), nomogram, and calibration were conducted to verify and evaluate the risk signature. Gene set enrichment analyses (GSEA) in the risk groups were used to figure out the closely correlated pathways with the risk group. The role of risk score has been further explored in the homologous recombination repair deficiency (HRD), BRAC1/2 gene mutations and tumor mutation burden (TMB). A risk signature with 4 CD8TEXGs in OC was finally built in the TCGA database and further validated in large GEO cohorts. The signature also demonstrated broad applicability across various types of cancer in the pan-cancer analysis. The high-risk score was significantly associated with a worse prognosis and the risk score was proven to be an independent prognostic biomarker. The 1-, 3-, and 5-years ROC values, nomogram, calibration, and comparison with the previously published models confirmed the excellent prediction power of this model. The low-risk group patients tended to exhibit a higher HRD score, BRCA1/2 gene mutation ratio and TMB. The low-risk group patients were more sensitive to Poly-ADP-ribose polymerase inhibitors (PARPi). Our findings of the prognostic value of CD8TEXGs in prognosis and drug response provided valuable insights into the molecular mechanisms and clinical management of OC.

**Supplementary Information:**

The online version contains supplementary material available at 10.1186/s40001-024-01948-8.

## Introduction

Ovarian cancer (OC) was a formidable disease and ranks as the fifth leading cause of cancer-related deaths of women worldwide. It was widely acknowledged as the most lethal gynecological cancer due to its high mortality rate, with nearly 13,270 deaths and over 19,710 new cases estimated in the US in 2023 [1]. The reason for death was largely due to a lack of specific symptoms and effective biomarkers for early detection [2]. Approximately 66% of patients were diagnosed at an advanced stage, and the 5-years overall survival (OS) rate was less than 50% [3, 4]. The most common treatment approach was based on conservative surgery, commonly combined with chemotherapy [5]. As precision medicine continues to advance, panel testing for homologous recombination repair deficiency (HRD) and BRCA1/2 gene mutations has emerged as an important tool for optimizing the use of Poly-ADP-ribose polymerase inhibitors (PARPi) and improving patient outcomes, even in the most advanced stages of OC. Such testing allowed for more precise identification of patients who were likely to benefit from PARPi, leading to more targeted and effective treatment strategies [6, 7]. Despite notable progress in treatment options for OC, such as the utilization of diverse therapy combinations that have contributed to certain reductions in OC-related mortality, patient outcomes continue to be predominantly unfavorable. Consequently, the imperative and indispensable undertaking of developing novel prognostic signatures and molecular biomarkers emerged, aiming to enhance patient outcomes and provide valuable insights for the implementation of more precise and efficacious treatment strategies.

The differentiation of CD8+T cells was a highly regulated process, primarily encompassing the naïve, effector, and memory states. Cytotoxic CD8+T cells played a pivotal role in eradicating chronic infections and malignant cells, thereby offering durable protective immunity [8, 9]. Nonetheless, when exposed to prolonged antigen stimulation, foreign antigens frequently became difficult to eliminate, leading to the emergence of a state known as CD8+T cell exhaustion. Exhausted CD8+T cells were marked by diminished secretion of effector cytokines, impaired proliferative capacity and persistence, as well as the expression of inhibitory receptors on their cell surface. These factors collectively contributed to a reduction in the effectiveness of T cell-mediated immunity [10, 11]. Recent advancements in single-cell technologies and genome-wide epigenetic profiling have provided valuable insights into the programming of exhausted CD8+T cells. These insights have opened up new avenues for the development of therapeutic strategies for cancer. Although some studies have investigated the role of immune cells in OC. For instance, a previous single-cell RNA-seq (scRNA-seq) identified two different immune patterns in OC [12]. Moreover, another OC study implicated ascites in remodeling the ecosystems of primary and metastatic tumors in OC [13]. An immune-related gene signature for risk stratification and prognosis prediction in OC [14]. However, the significance of exhausted CD8+T cells in OC prognosis and treatment remains unclear and there was no CD8TEXGs signature has been built in OC. In this study, our objective was to obtain a comprehensive understanding of the prognostic significance of CD8TEXGs in OC by utilizing bulk and single-cell sequencing datasets. We evaluated various clinical features, including OS, progress-free survival (PFS) and disease-free survival (DFS), HRD, as well as the effectiveness of PARPi, to compare outcomes between subpopulations with high-risk and low-risk scores.

## Materials and methods

### Data acquisition

We obtained RNA-seq gene expression data in transcripts per million (TPM) values, clinical information, and masked annotated somatic mutation datasets of OC from TCGA (https://portal.gdc.cancer.gov/). scRNA-seq data (GSE130000) [15] and validation datasets for prognosis (GSE102073, GSE140082, GSE165808, GSE17260, GSE19829, GSE26193, GSE26712, GSE30161, GSE32062, GSE32063, GSE51088, GSE53963, GSE63885, GSE73614, GSE9891) were obtained from the GEO database (https://www.ncbi.nlm.nih.gov/geo/) [16, 17]. The TCGA TPM values were log2(x + 1) transformed. Datasets for pan-cancer analysis was sourced from the UCSC Xena database (https://xenabrowser.net/) [18].

### Comprehensive analysis of single-cell datasets and cell cluster annotation

Raw count matrix of scRNA-seq data was downloaded from The Tumor Immune Single Cell Hub 2 (TISCH2) database (http://tisch.comp-genomics.org/). TISCH2 applied the MAESTRO workflow to process all the collected datasets. This processing included quality control, batch effect removal, cell clustering, differential gene expression analysis, and cell type annotation [19]. In our study, we initially obtained CD8TEXGs from TISCH2 using the following criteria: |log2FC|> 1 and adjusted p value < 0.05. The re-analysis of the scRNA-seq dataset was done by using the R package “Seurat” (v4.1.1) [20]. By default, “LogNormalize” function was used to normalize the feature expression measurements for each cell by the total expression, multiply this by a scale factor (10,000 by default), and log-transform the result. The uniform manifold approximation and projection (UMAP) and clustering results were acquired from TISCH2 database. Cell types were re-annotated on the basis of the known marker genes. For visualization purposes, dot plot was utilized. To evaluate the metabolic characteristics of different cell subtypes, the metabolic scores were calculated using the R package “scMetabolism”. This was achieved by employing the AUCell method in the reactome pathway [21]. The results derived from the scMetabolism analysis were integrated and visualized using dot plot, presenting a comprehensive perspective of the metabolic landscape among various cell subtype clusters.

### Construction of CD8TEXGs risk score signature

Using the TCGA dataset as internal dataset, internal validation randomize the data into training and testing sets at a 1:1 ratio firstly. And training set was used to select variables and construct model, testing set used to validate the result. To identify genes associated with OS in OC patients, we performed a series of analyses including univariate Cox regression, LASSO regression, and multivariate Cox regression. These analyses allowed us to identify four CD8TEXGs that demonstrated significant associations with OS. Subsequently, based on their expression levels and corresponding multivariate Cox regression coefficients, we calculated the risk score using the following formula:

Risk score = ∑multivariate Cox regression coefficient (gene x) * gene expression value (gene x). External GEO datasets were used to validate the model in OS, PFS, and DFS. Cutoff risk score value and subsequently divided the patients into high-risk and low-risk subgroups by median value.

### Nomogram and calibration

To assess the prognostic value of the risk score over time in the entire TCGA dataset, we performed ROC analysis. Additionally, we investigated the role of the risk score in different clinical subgroups, including age, grade, stage, and tumor residual size. To provide a comprehensive predictive tool, we constructed a nomogram using multivariate Cox regression analysis. This nomogram integrated both clinical information and the risk score (utilizing the “regplot” package in R). Furthermore, calibration curves were employed to evaluate the accuracy of the nomogram.

### Functional enrichment analysis

To identify highly relevant KEGG and HALLMARK pathways between the high-risk and low-risk subgroups, we employed the GSEA v4.3.2 tool from the MSigDB database (http://software.broadinstitute.org/gsea/msigdb/). Our selection criterion for pathway analysis was based on statistical significance, with thresholds set as false discovery rate (FDR) < 0.25 and Nominal p value < 0.05 [22, 23].

### Calculation of TMB, HRD scores

To quantify the TMB, we computed the mean number of mutations within the exonic region of the tumor genome, encompassing gene coding errors, base substitutions, insertions, and deletions. The dataset containing information on BRCA1 and BRCA2 mutations was acquired from the masked annotated somatic mutation dataset, if the sample had a mutation in gene BRCA1 or BRCA2, we categorized it as mutated BRCA1/2 sample. Regarding the assessment of the HRD scores, we utilized the scores derived from a prior study [24]. To assess the differences between the low-risk and high-risk subgroups, we conducted a Wilcoxon test. Furthermore, we evaluated the immune cell infiltration using the TIP database (http://biocc.hrbmu.edu.cn/TIP/) [25].

### Drug sensitivity analysis

The response to PARPi was determined by calculating the half-maximal inhibitory concentration (IC50) using data from the Genomics of Drug Sensitivity in Cancer (GDSC) database (https://www.cancerrxgene.org/), specifically through the utilization of the R package “pRRophetic” [26].

### Quantitative real-time PCR

Total RNA was extracted from ISOE, SKOV3, and A2780 cell lines using the Trizol. Subsequently, the RNA was reverse transcribed into complementary DNA (cDNA). Primers for the PCR reactions were designed and obtained from the Genewiz Company. For the real-time PCR analysis, the cDNA was utilized as the template, and the PCR reaction was performed using the QuantStudio™ 7 Flex System. The primer sequences employed in the analysis are provided in (Additional file 1: Table S1).

### Statistical analysis

All statistical analyses were performed using R software, specifically versions 4.2.2. A p value < 0.05 was considered statistically significant unless stated otherwise. Ns, *, **, ***, and **** stood for p value > 0.05, p value ≤ 0.05, p value ≤ 0.01, p value ≤ 0.001, and p value ≤ 0.0001, respectively. Survival analysis was conducted using the R packages “survival” and “survminer”. The Wilcoxon test was employed for comparing two groups, while the Kruskal–Wallis test was used for comparing more than two groups.

## Results

**Fig. 1:**
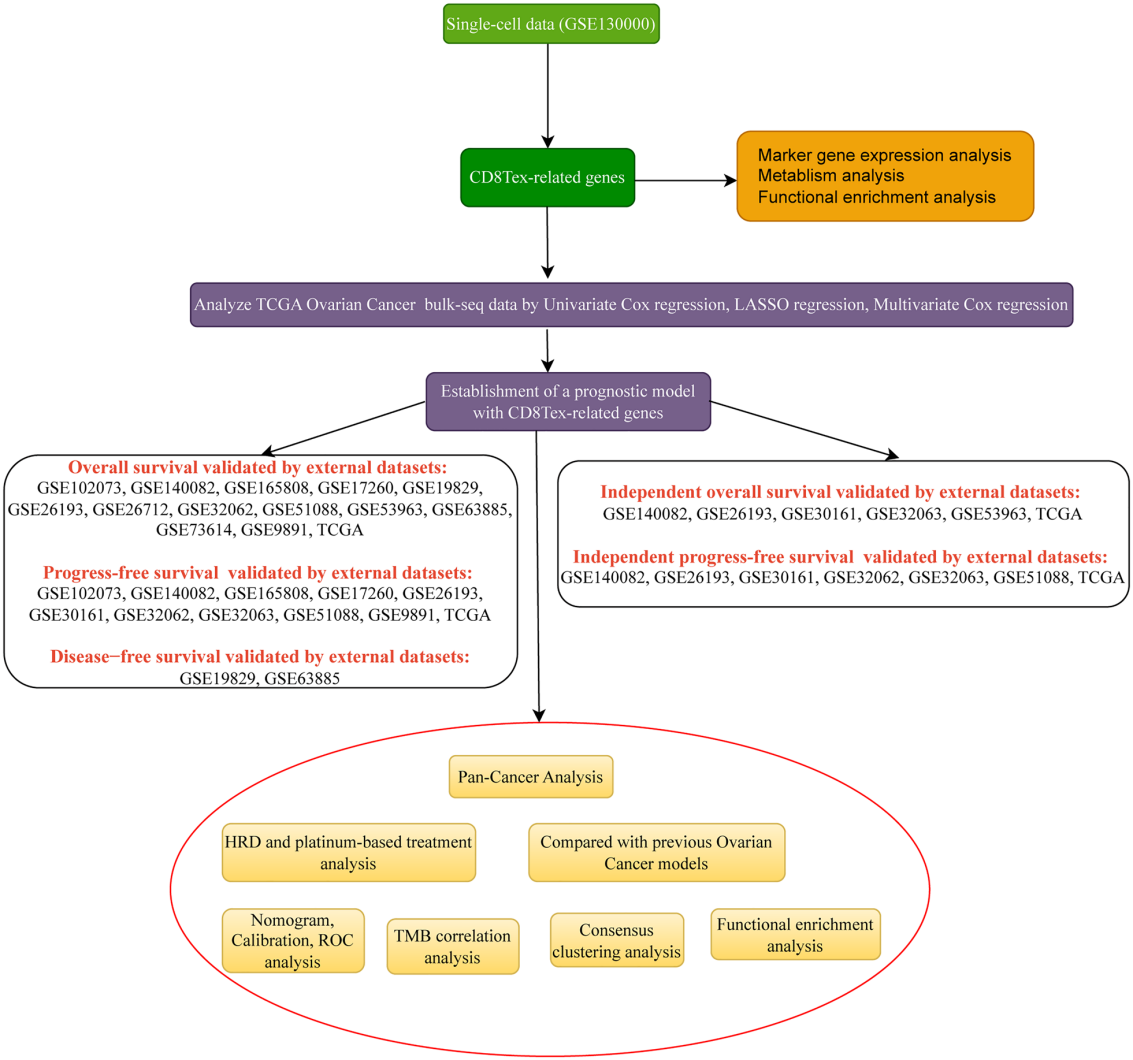
The study included a specific workflow for data analysis, represented by a workflow diagram, which outlined the sequential steps involved in the analysis of the collected data

The complete workflow of this study is depicted in Fig. 1.

### Analysis of OC single-cell sequencing data

**Fig. 2:**
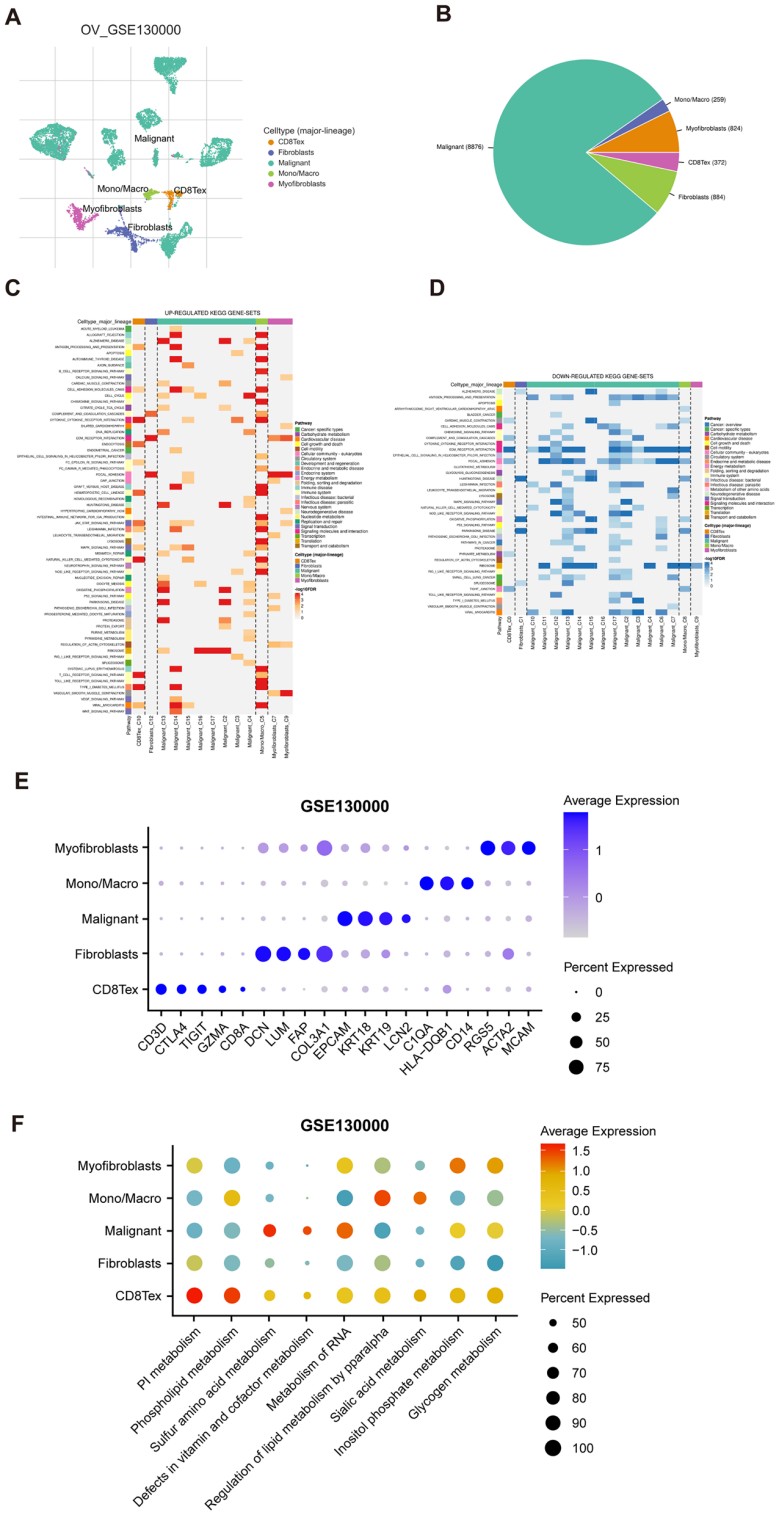
OC single-cell data analysis based on the GSE13000 dataset. **A** The UMAP plots with cells colored by cell type were displayed. **B** The pie plot showed the cell number distribution of each cell type. **C**The heatmap showed functionally enriched up-regulated KEGG pathways identified based on differential genes in each cell type. **D** The heatmap showed functionally enriched down-regulated KEGG pathways identified based on differential genes in each cell type. **E** Gene expression of different classical cell type markers. **F** The single-cell metabolic features of cell subsets

We utilized TISCH2 database to obtain the scRNA-seq datasets, specifically GSE130000, which was generated using the Drop-seq platform. The dataset was re-analyzed using the R package Seurat. As depicted in Fig. 2A, B, our analysis revealed that exhausted CD8+T cells represented the largest proportion of immune cells in the dataset. Notably, the GSEA analysis of KEGG pathways demonstrated that exhausted CD8+T cells were significantly enriched in cytokine–cytokine receptor interaction, nature killer cell-mediated cytotoxicity, T cell receptor signaling pathway, ECM receptor interaction (Fig. 2C, D). These findings suggested that exhausted CD8+T cells played a critical role in OC-related immune pathways and warranted further investigation. We have provided a list of markers for each cell type in Additional file 1: Table S2, and their expression patterns are illustrated in Fig. 2E. It was easy to find the classical marker, CD3D, CTLA4, TIGHT, GZMA, and CD8A were mainly expressed on CD8Tex (exhausted CD8+T) subset (Fig. 2E). Furthermore, we examined the metabolic status of different cell type clusters. The analysis revealed that CD8Tex cells exhibited enrichment in phospholipid metabolism and pi metabolism pathways within the GSE130000 dataset (Fig. 2F).

### Development and validation of prognostic signatures associated with CD8TEXGs in OC

**Fig. 3:**
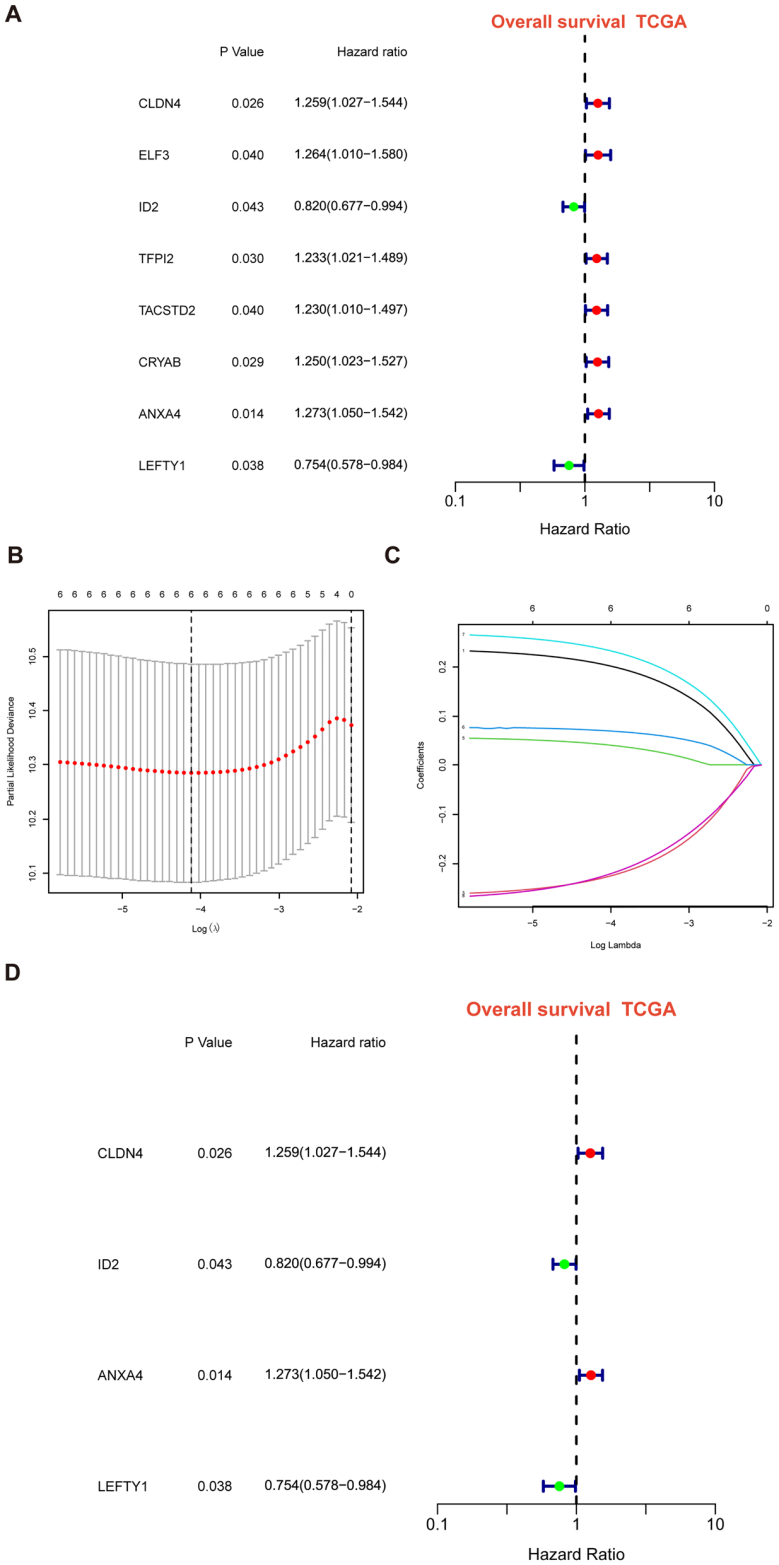
Establishing a signature of exhausted CD8+T cells-related genes in OC. **A** Prognosis-associated genes were extracted by univariate Cox regression analysis. **B** Ten-fold cross-validation for variable selection in LASSO regression analysis. **C** LASSO coefficient profile of candidate genes. **D** Prognosis-associated genes were extracted by multivariate Cox regression analysis

**Fig. 4:**
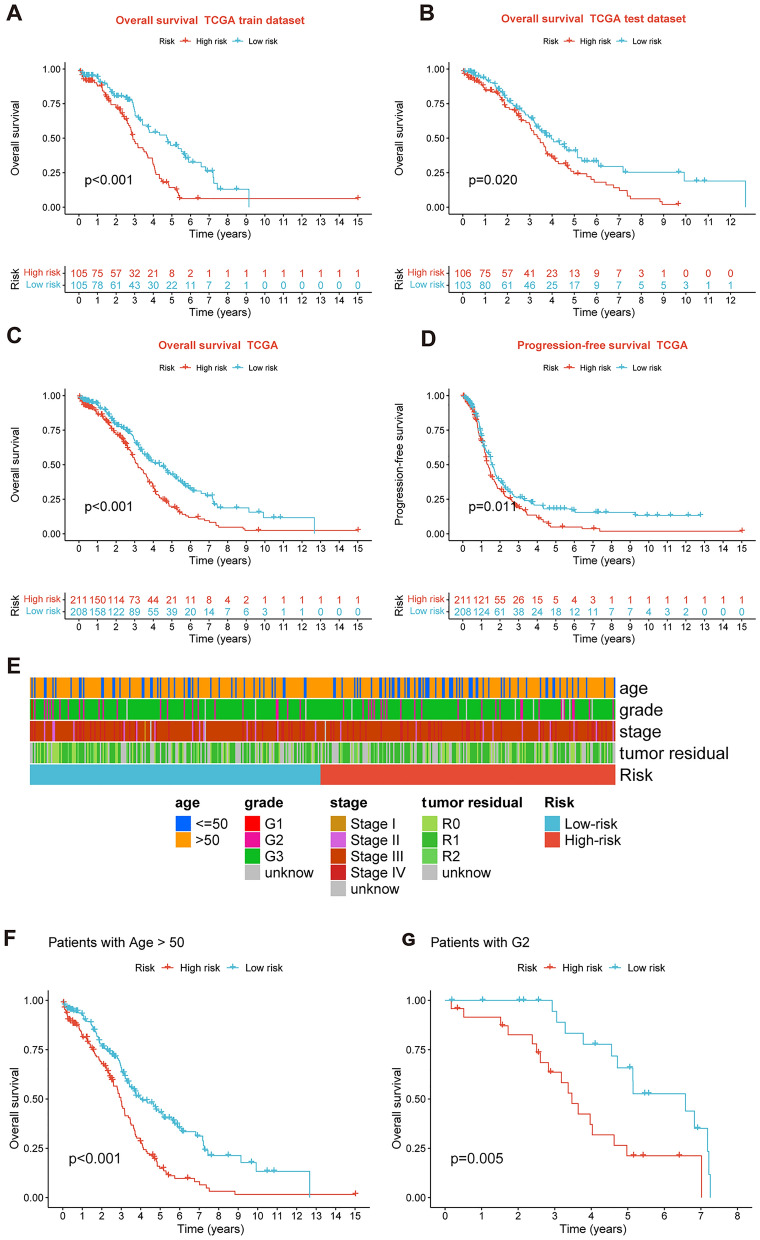

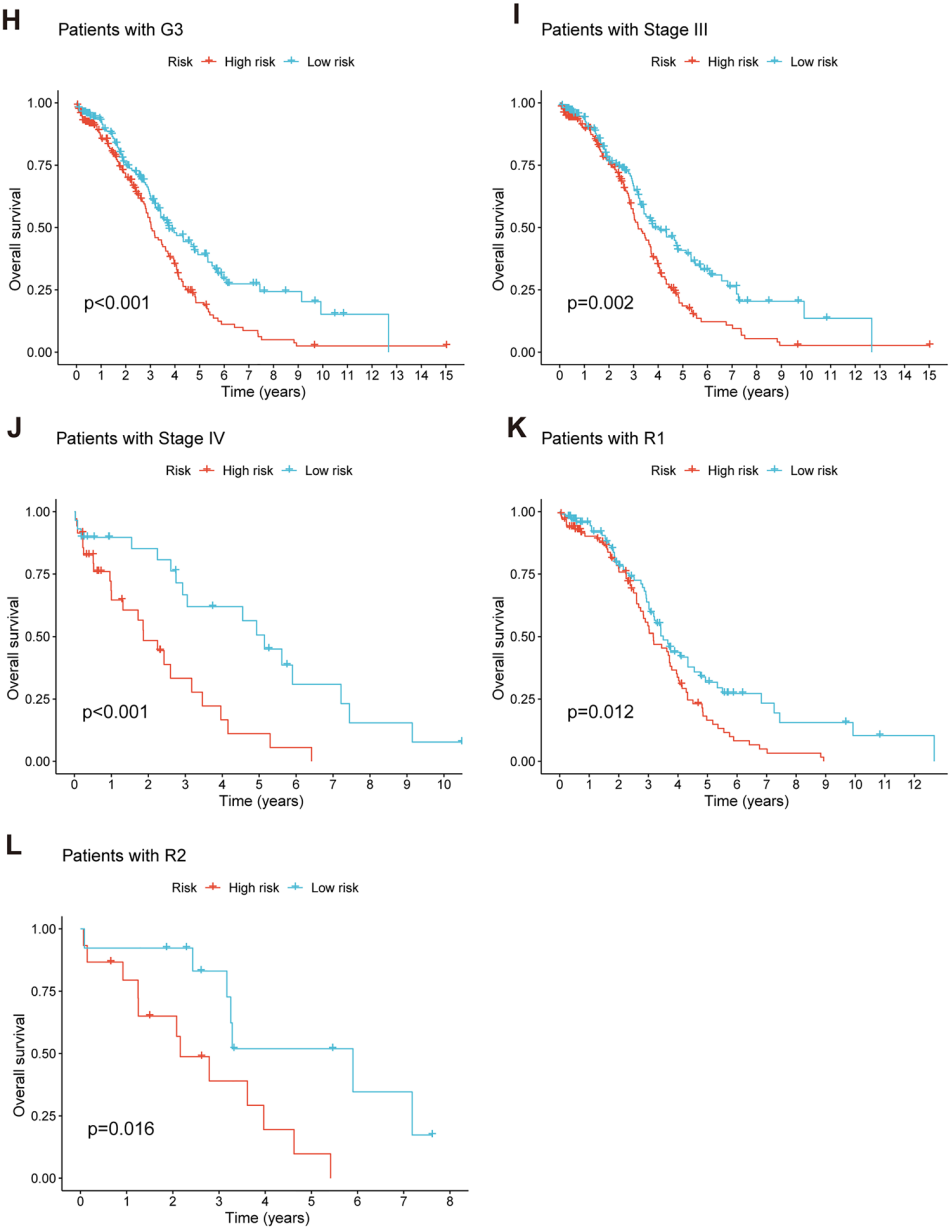
Prognosis value of the four exhausted CD8+T cells-related genes signature in the train, test, and whole TCGA datasets. **A**–**C** OS analysis in the train, test, and whole TCGA datasets. **D** PFS in the whole TCGA dataset. **E** Clinical information comparison between the high-risk and low-risk groups. **F**–**L** The prognostic value was stratified by age, stage, and tumor residual size between high-risk and low-risk subgroups in the whole TCGA dataset

**Fig. 5:**
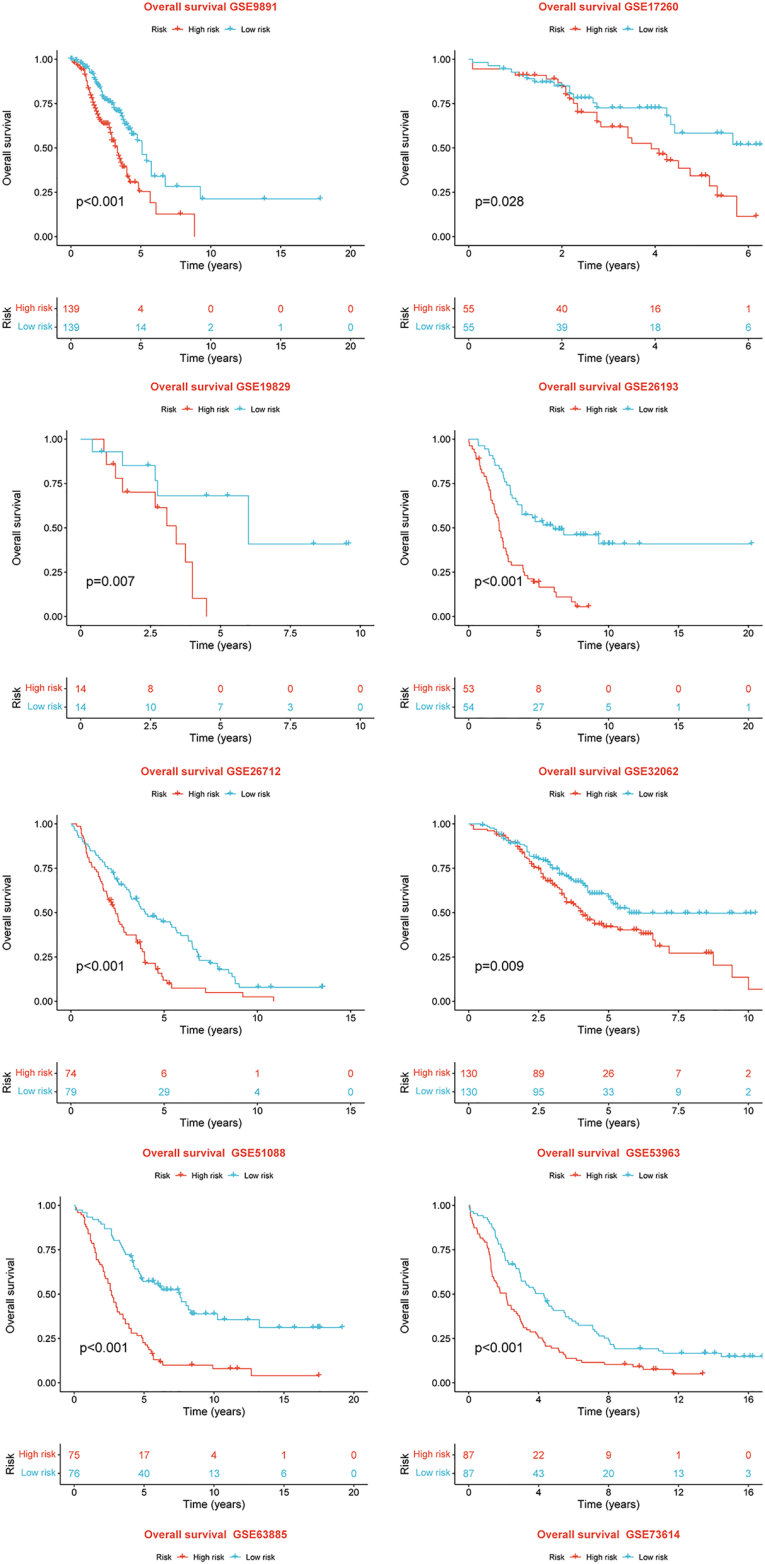

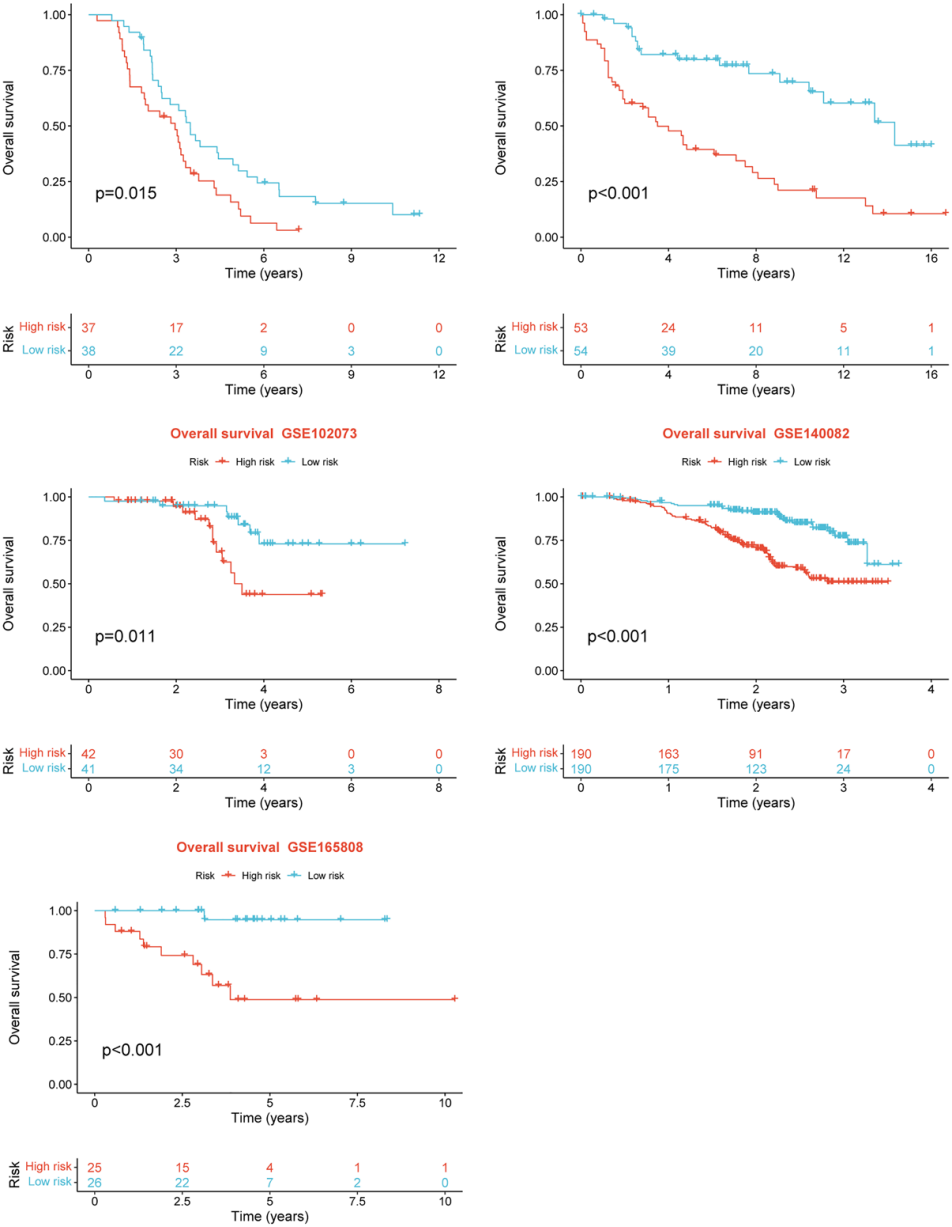
Thirteen external validation datasets of the exhausted CD8+T cells-related genes signature in OS

**Fig. 6:**
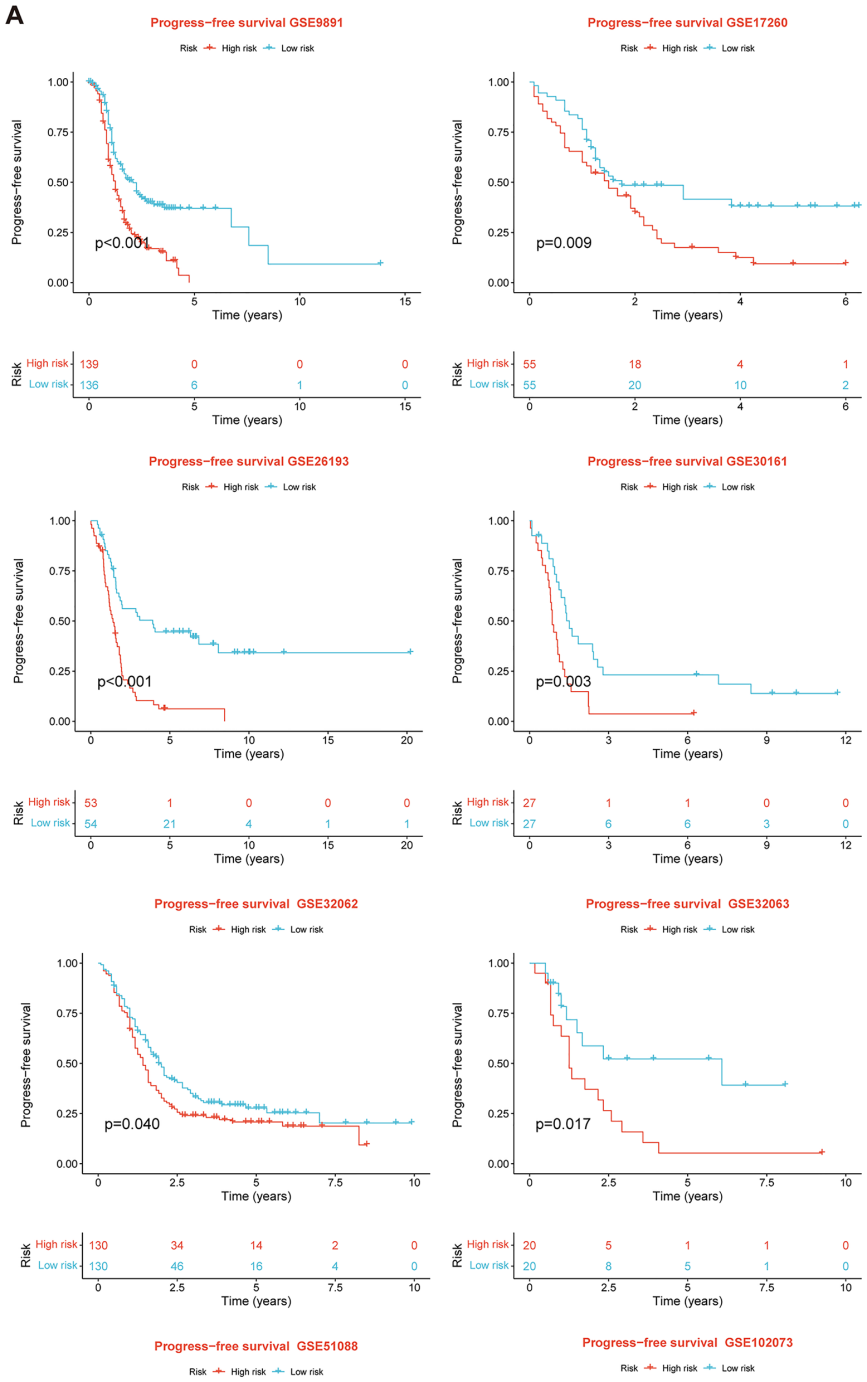

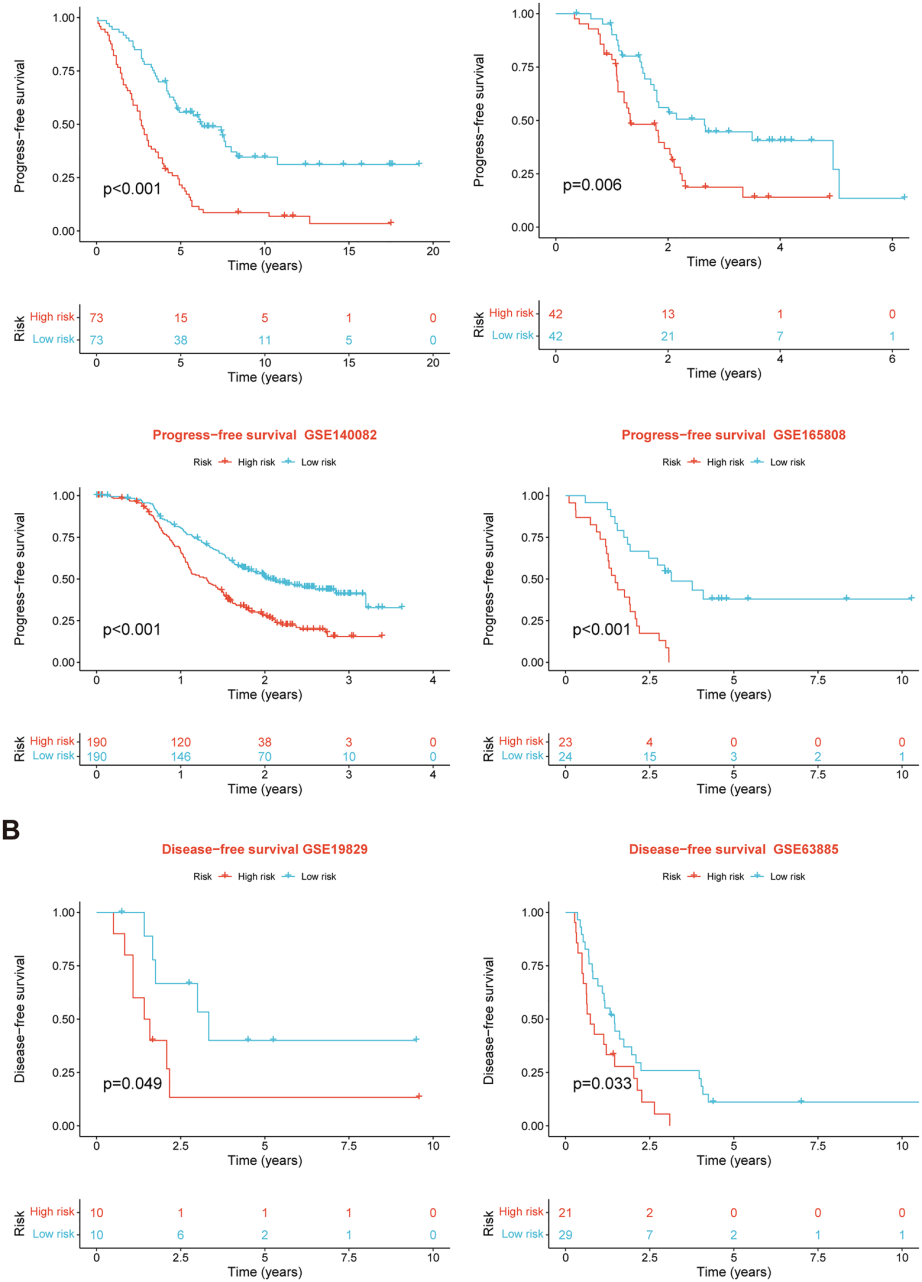
External validation datasets of the exhausted CD8+T cells-related genes signature in PFS and DFS. **A** In PFS. **B** In DFS

**Fig. 7:**
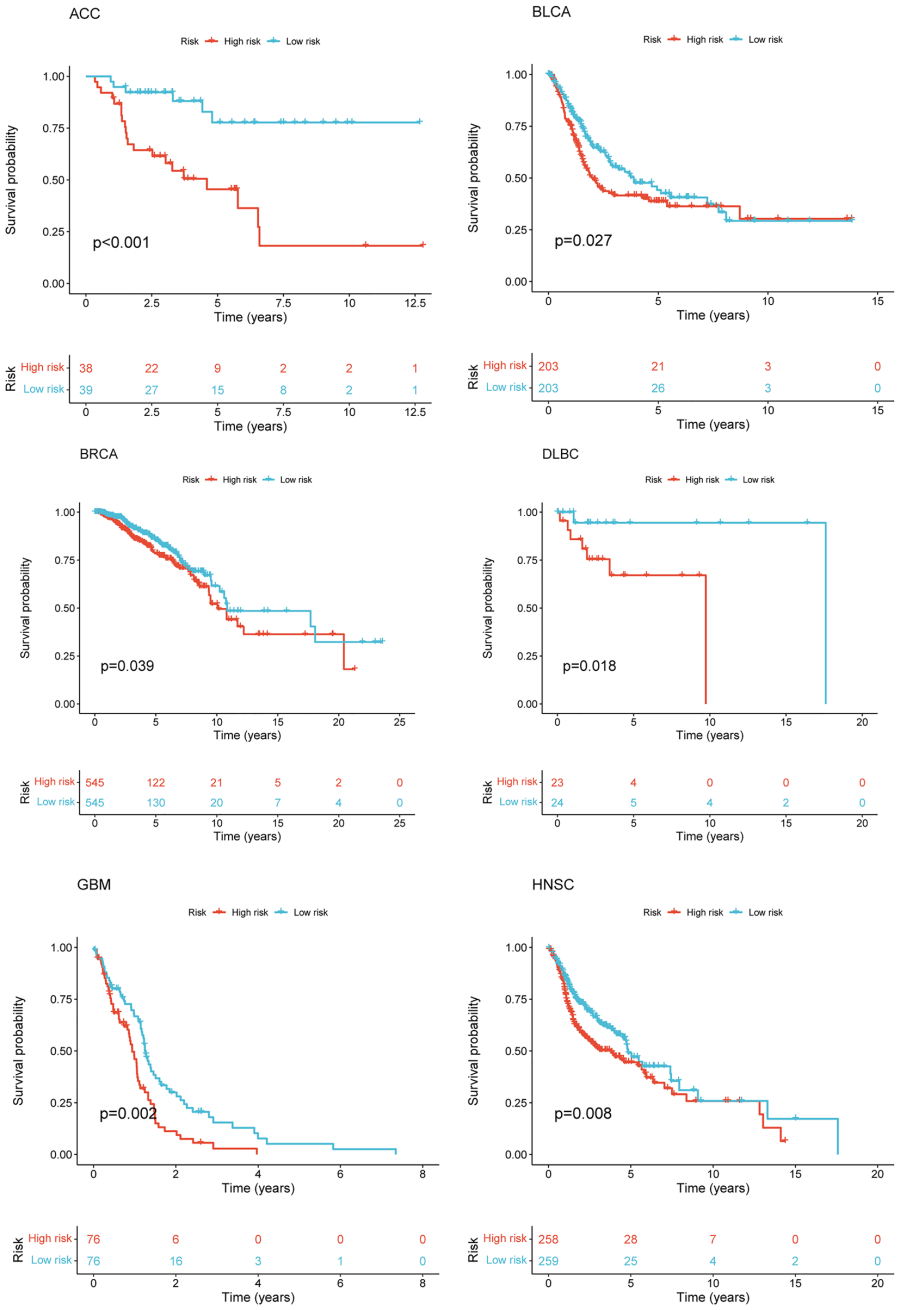

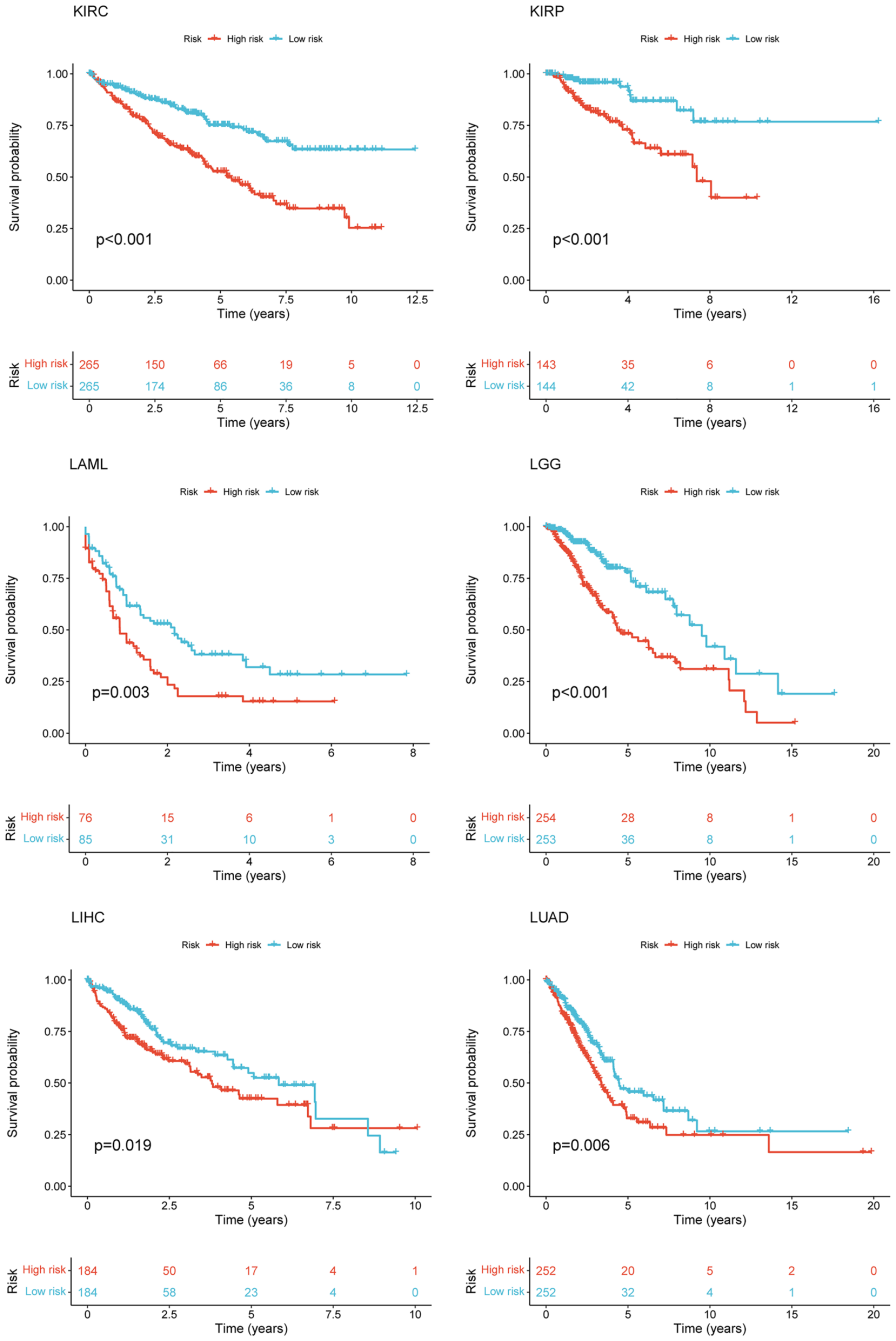
Pan-cancer analysis of the exhausted CD8+T cells-related genes signature

**Fig. 8:**
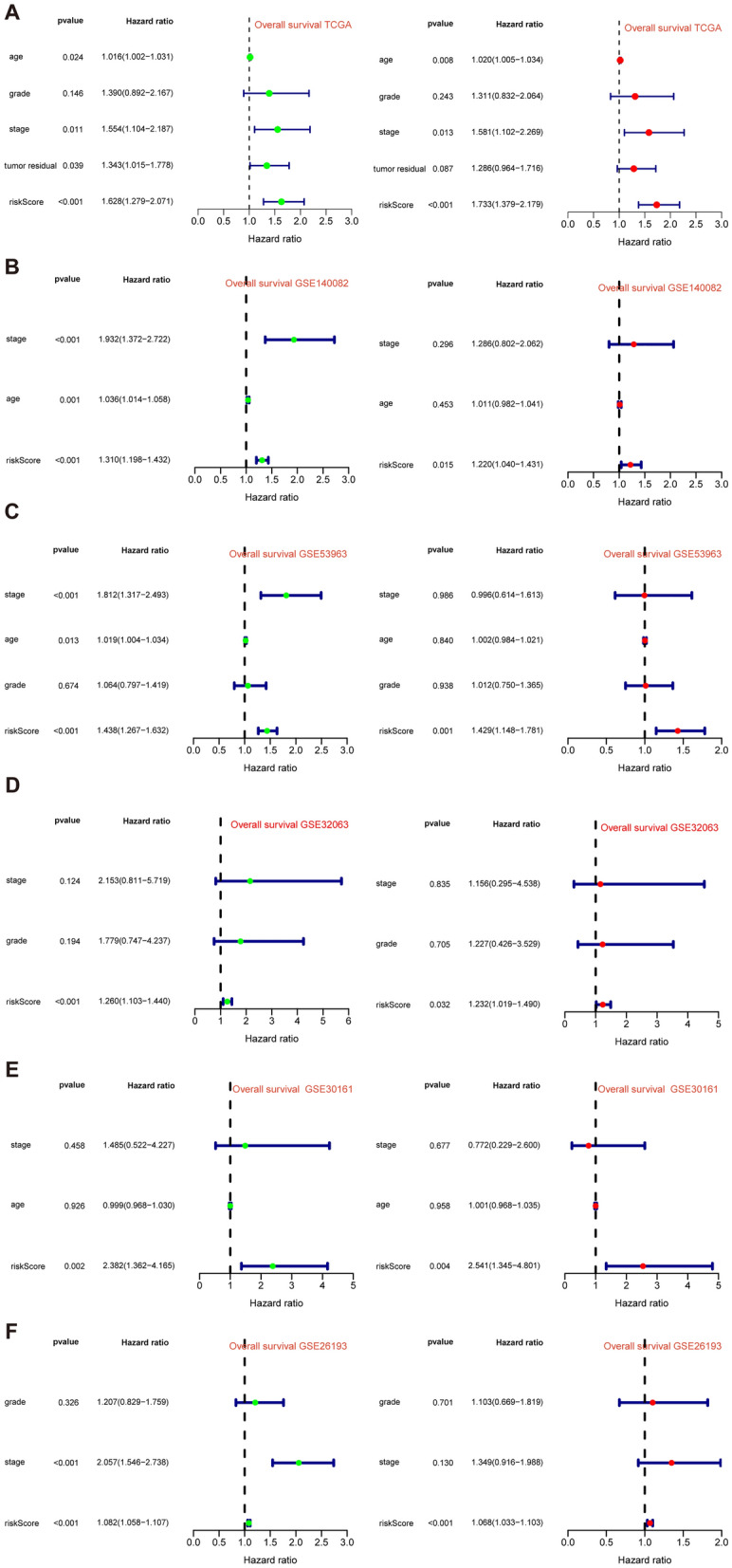
Risk score as an independent prognostic factor in different datasets of OS. **A** Univariate and multivariate Cox regression analysis in the TCGA. **B**–**F** Similar analyses in the other datasets

**Fig. 9:**
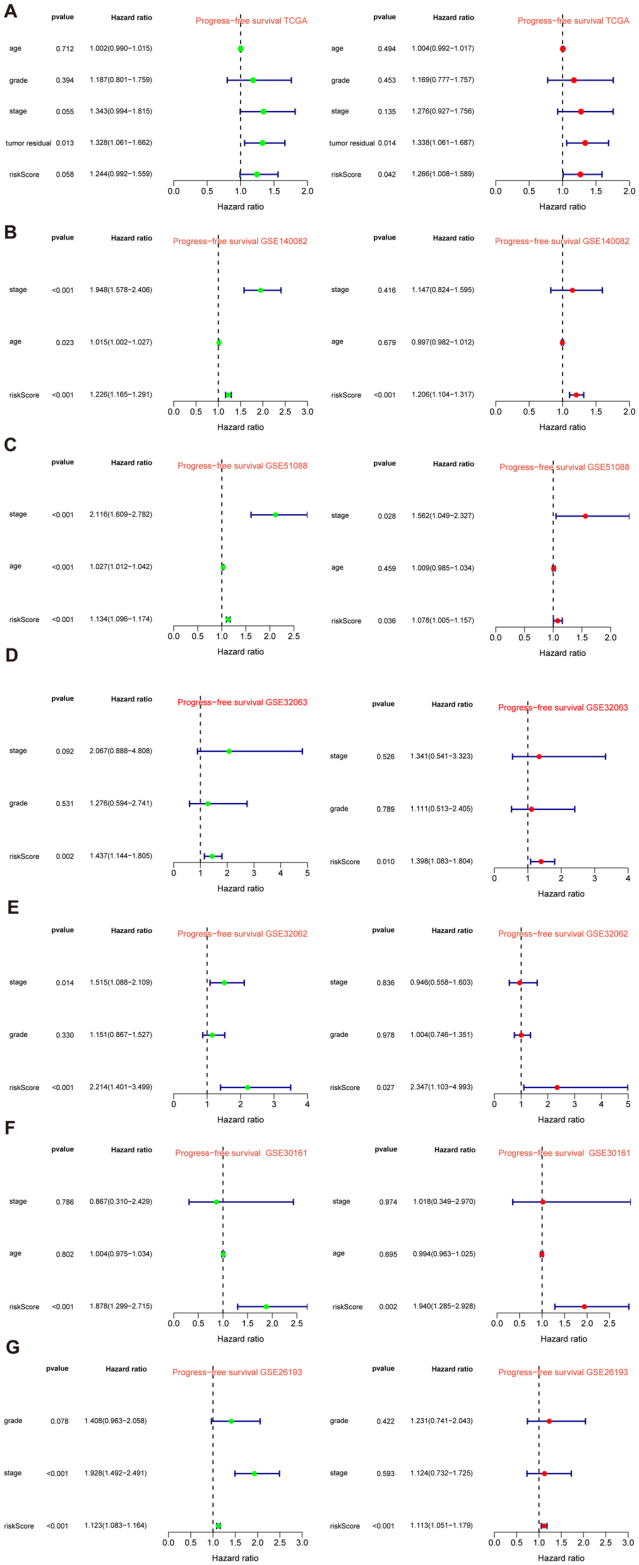
Risk score as an independent prognostic factor in different datasets of PFS. **A** Univariate and multivariate Cox regression analysis in the TCGA. **B**–**G** Similar analyses in the other datasets

**Fig. 10:**
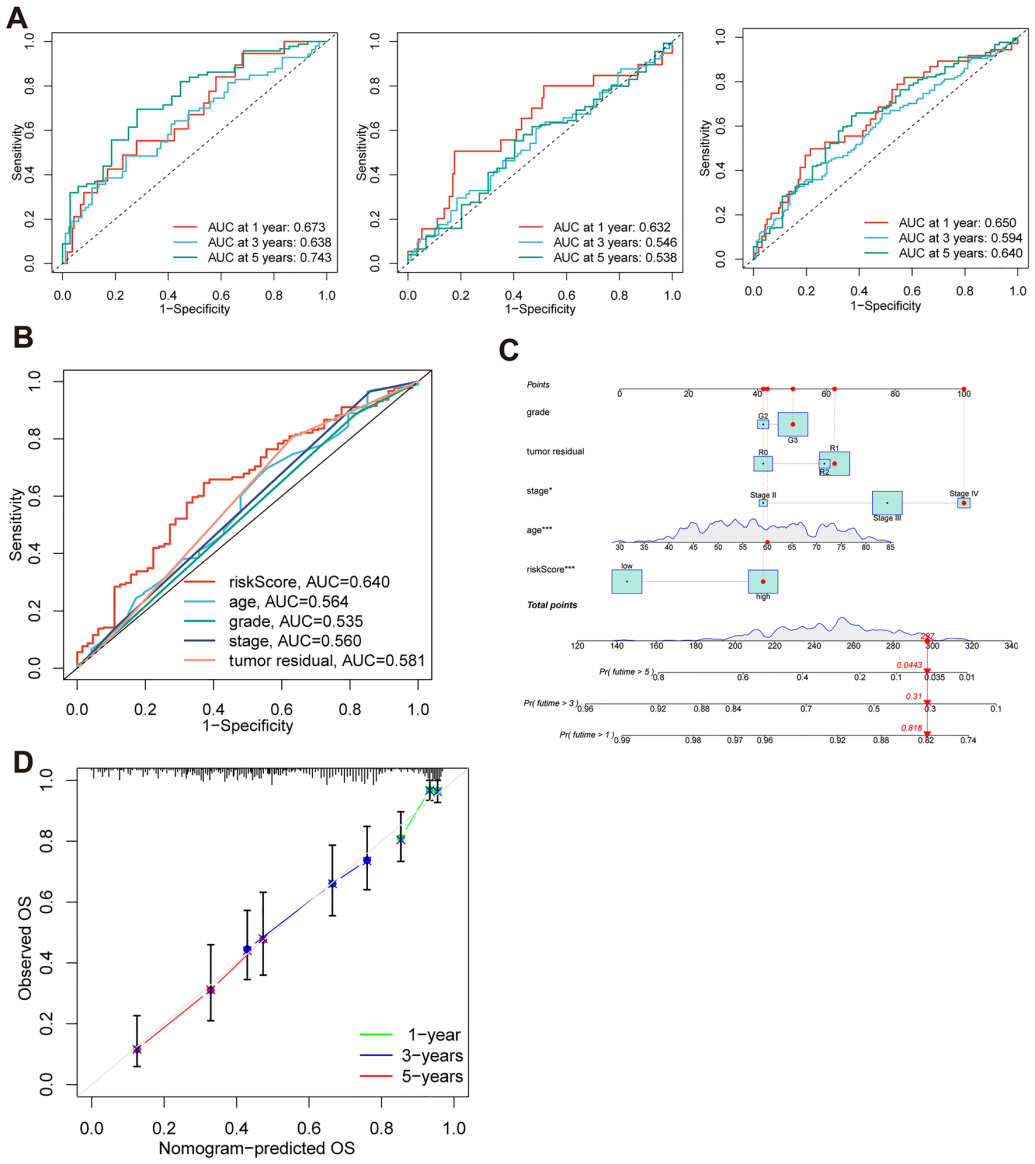
ROC, nomogram, and calibration curves for evaluating risk score and OS prediction. **A** ROC curves were generated to evaluate the predictive accuracy of the risk score at 1, 3, and 5 years in the train, test, and whole datasets. **B** ROC curves were generated to compare the power of risk score and other clinical features. **C** A nomogram was developed, incorporating the risk score, age, grade, stage, and tumor residual size, to predict the probability of 1-, 3-, and 5-year OS. **D** Calibration curves were analyzed to assess the calibration performance of the nomogram for 1-, 3-, and 5-year OS predictions

After intersecting the genes in the scRNA-seq GEO dataset and the bulk-seq TCGA dataset, a total of 132 CD8TEXGs were identified. The list of these genes could be found in Additional file 1: Table S3. To identify significant genes associated with OS, we initially performed univariate Cox regression analysis, resulting in the identification of eight genes. The list of these genes could be found in Additional file 1: Table S4, and the forest plot was shown in Fig. 3A. The internal validation TCGA dataset was divided into train and test datasets at a 1:1 ratio. In order to refine the gene list and create a more robust model, we further employed the LASSO algorithm using the optimal lambda value, followed by multivariate Cox regression analyses (Fig. 3B–D). Ultimately, four genes were selected, and based on their expression, a risk score model was generated for the final analysis. The risk score was calculated as follows: risk score = (0.262 * CLDN4 expression) + (− 2.82 * ID2 expression) + (0.295 * ANXA4 expression) + (− 0.297 * LEFTY1 expression). Based on the median risk score, patients with OC were classified into high-risk and low-risk subgroups within the TCGA dataset. The findings consistently demonstrated that the high-risk group exhibited a poorer prognosis across the train, test, and whole datasets (Fig. 4A–C). Furthermore, we observed that the PFS also exhibited significant differences between the high-risk and low-risk subgroups in the TCGA whole dataset (Fig. 4D). To account for potential discrepancies in prognosis resulting from variations in clinical data, we compared clinical features such as age, grade, stage, and tumor residual size between the high-risk and low-risk subgroups within the TCGA whole dataset. The analysis revealed no significant differences (Fig. 4E), and the statistical comparison results can be found in Additional file 1: Table S5. Detailed clinical information is provided in Additional file 1: Table S6. Hence, our findings provided evidence that the disparity in prognosis could be attributed to our risk signature rather than an imbalance in the grouping of clinical data. Furthermore, we assessed the performance of the risk score across different clinical characteristics to expand its potential applications. Age > 50 years, G2 and G3, stage III and stage IV, R1 and R2 were significant prognostic between high-risk and low-risk subgroups in the TCGA whole dataset (Fig. 4F–L). The majority of the aforementioned analyses primarily relied on the TCGA dataset. To validate the accuracy and robustness of our model, we sought external datasets for validation purposes. Notably, the overall OS analyses conducted on GEO datasets, GSE102073, GSE140082, GSE165808, GSE17260, GSE19829, GSE26193, GSE26712, GSE32062, GSE51088, GSE53963, GSE63885, GSE73614, and GSE9891, consistently showed significant results (Fig. 5). Similarly, the PFS analyses on GEO datasets, GSE102073, GSE140082, GSE165808, GSE17260, GSE26193, GSE30161, GSE32062, GSE32063, GSE51088, and GSE9891 also exhibited significant results (Fig. 6A). Additionally, the DFS analyses showed significant results on GSE19829 and GSE63885 (Fig. 6B). We also discovered that our model had broad applicability for OS to other cancer types in the pan-cancer analysis, especially for cancers with high incidence and mortality rates. These include adrenocortical carcinoma (ACC), bladder urothelial carcinoma (BLCA), breast invasive carcinoma (BRCA), diffuse large B-cell lymphoma (DLBC), glioblastoma multiforme (GBM), head and neck squamous cell carcinoma (HNSC), kidney renal clear cell carcinoma (KIRC), kidney renal papillary cell carcinoma (KIRP), acute myeloid leukemia (LAML), brain lower grade glioma (LGG), liver hepatocellular carcinoma (LIHC), and lung adenocarcinoma (LUAD) (Fig. 7). To evaluate whether the risk score could function as an independent prognostic factor, we conducted an integrated analysis by combining clinical features with our pre-calculated risk score. The results of the univariate and multivariate Cox regression analyses indicated that the risk score was an independent factor significantly associated with OS in the datasets TCGA, GSE140082, GSE53963, GSE32063, GSE30161 and GSE26193 (Fig. 8A–F). The results of the univariate and multivariate Cox regression analyses demonstrated that the risk score was a significant factor for PFS in the TCGA, GSE140082, GSE51088, GSE32063, GSE32062, GSE30161 and GSE26193 datasets (Fig. 9A–G). To assess the predictive ability of the risk signature, we performed ROC analysis. The values at 1, 3, and 5 years for predicting OS were as follows: 0.673, 0.638, and 0.743 in the TCGA train dataset, 0.632, 0.546, and 0.538 in the TCGA test dataset, and 0.650, 0.594, and 0.640 in the TCGA whole dataset, respectively (Fig. 10A). Furthermore, we observed that the risk score demonstrated a higher area under the ROC Curve (AUC) compared to other clinical features in the TCGA whole dataset (Fig. 10B). This finding implied the reliability and prioritization of the risk score as an independent prognostic factor.

### Analyzing and estimating nomogram

To enhance the clinical utility of the risk model and facilitate the prediction of survival risk in OC patients, we developed a nomogram utilizing the risk score and four other critical clinical features in the TCGA cohort. This nomogram allowed for the calculation of an integrated point for each patient, enabling the accurate quantification of survival rates (Fig. 10C). To assess the performance of the nomogram, calibration curves were generated. These curves demonstrated a close alignment between the actual OS rates at 1, 3, and 5 years and the rates estimated by the nomogram (Fig. 10D).

### Functional enrichment analysis of the 4 CD8TEXGs risk model

**Fig. 11:**
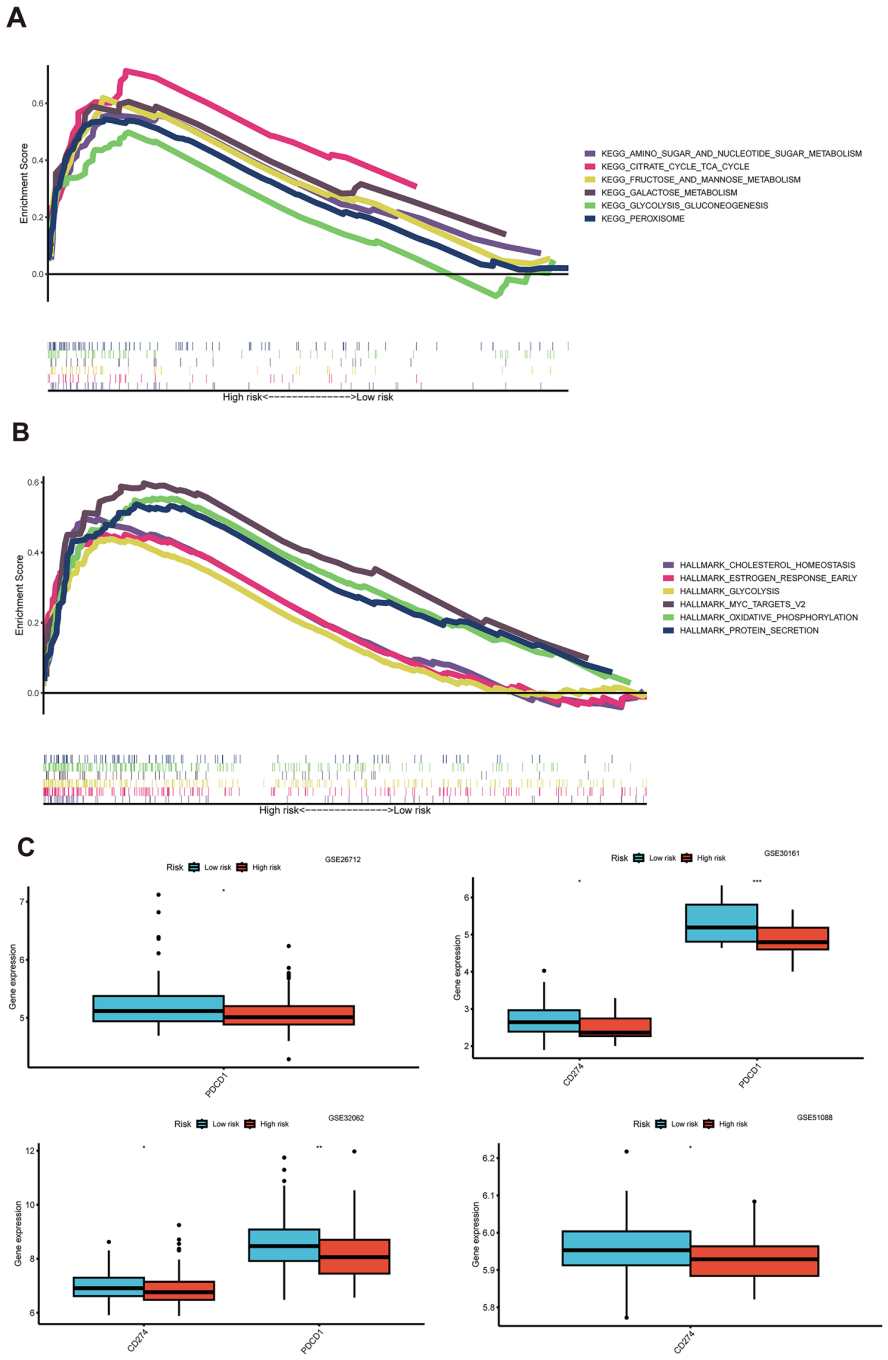

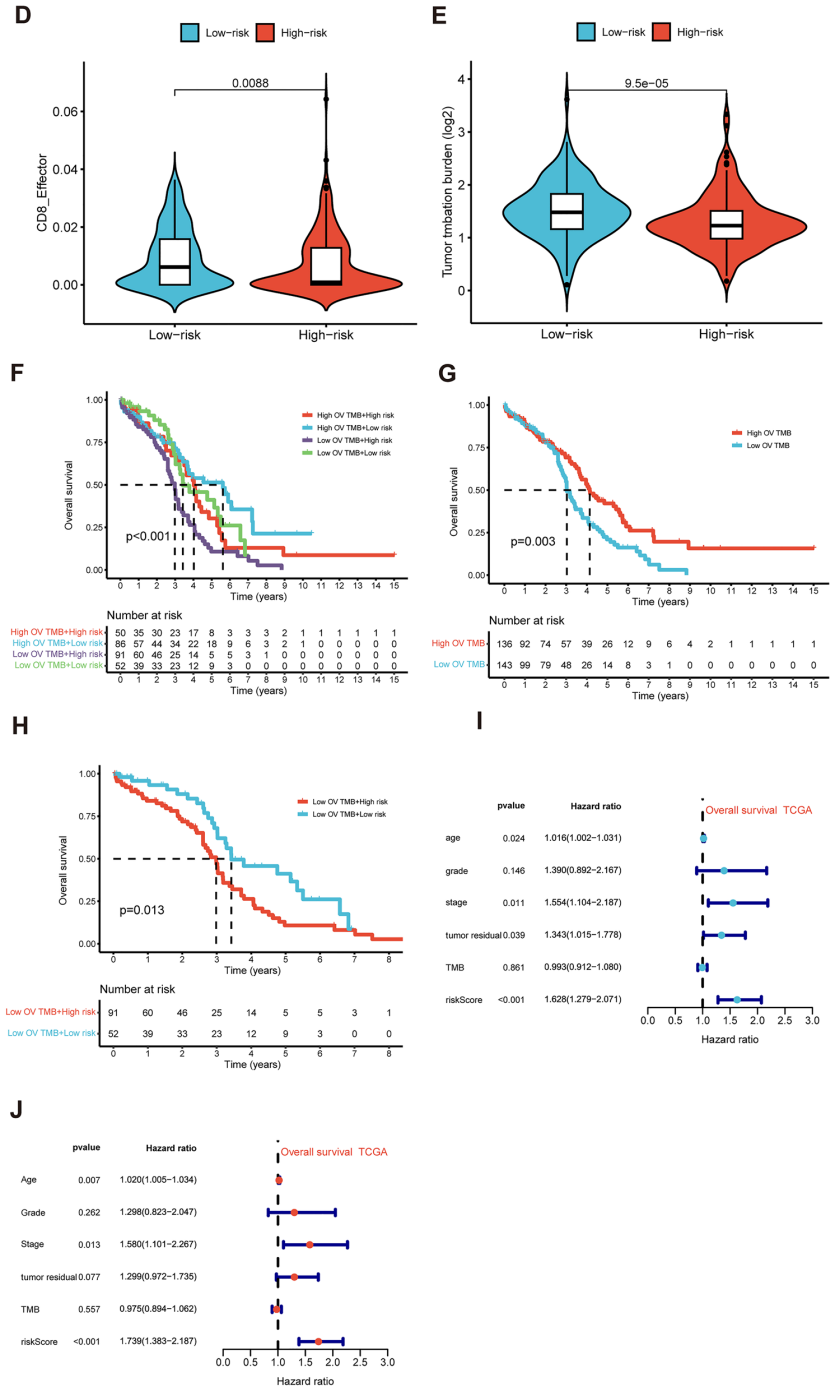
GSEA and TMB comparison between the Risk Groups. **A** Highly enriched KEGG terms in the high-risk group. **B** Highly enriched Hallmark pathways in the high-risk group (pvalue < 0.05, FDR < 0.25). **C** Immune checkpoint genes expression between low-risk and high-risk groups in different datasets. **D** CD8T effector infiltration level difference between low-risk and high-risk groups. **E** TMB level difference between low-risk and high-risk groups. **F** OS was compared between the combination of different TMB and risk score levels. **G** OS was compared between the low-TMB and high-TMB groups. **H** OS was compared between the combinations of different risk score levels with low-TMB level. **I** Univariate Cox regression analysis was conducted to assess the prognostic significance of the risk score, along with age, stage, grade, and tumor residual size, TMB. **J** Multivariate Cox regression analysis was performed to determine the independent prognostic value of the risk score, age, stage, grade, and tumor residual size, TMB

To investigate the disparities in biological function between the high-risk and low-risk groups determined by the risk score, we utilized the GSEA software. GSEA was employed to analyze KEGG and HALLMARK gene sets across the entire TCGA dataset, comparing the high-risk and low-risk groups based on comprehensive gene information. The significant enriched KEGG terms in the low-risk group were KEGG AMINO SUGAR AND NUCLEOTIDE SUGAR METABOLISM, KEGG CITRATE CYCLE TCA CYCLE, KEGG FRUCTOSE AND MANNOSE METABOLISM, KEGG GALACTOSE METABOLISM, KEGG GLYCOLYSIS GLUCONEOGENESIS, et al. (Fig. 11A). The significant enriched HALLMARK terms in the low-risk group were HALLMARK CHOLESTEROL HOMEOSTASIS, HALLMARK ESTROGEN RESPONSE EARLY, HALLMARK GLYCOLYSIS, HALLMARK MYC TARGETS V2, HALLMARK OXIDATIVE PHOSPHORYLATION, HALLMARK PROTEIN SECRETION, et al. (Fig. 11B). Immune checkpoint blockade has emerged as a promising strategy for treating various cancers. We investigated the expression levels of key immunomodulators, including CD274 (PD-L1) or PDCD1 (PD-1). As illustrated in Fig. 11C, low-risk patients exhibited higher expression of these immune checkpoint molecules compared to high-risk patients in datasets GSE26712, GSE30161, GSE32062, and GSE51088. The CD8+T cell effector (CD8_Effector) infiltration level was found to be upregulated in the low-risk score group (Fig. 11D). TMB was found to be upregulated in the low-risk score group (Fig. 11E). Combined analysis of TMB and risk score, the results showed that there was a significant difference in OS between the high TMB plus high-risk, high TMB plus low-risk, low TMB plus high-risk, low TMB plus low-risk (Fig. 11F). The high TMB showed a better clinical outcome (Fig. 11G) and the low-risk group presented a better significant survival compared to the high-risk group in the low TMB group (Fig. 11H). When integrating clinic features, TMB and risk score into a Cox model, the results showed the risk score was still significant in both univariate and was an independent prediction factor in multivariate Cox regression analyses (Fig. 11I, J).

### The relationship between risk score and HRD

**Fig. 12:**
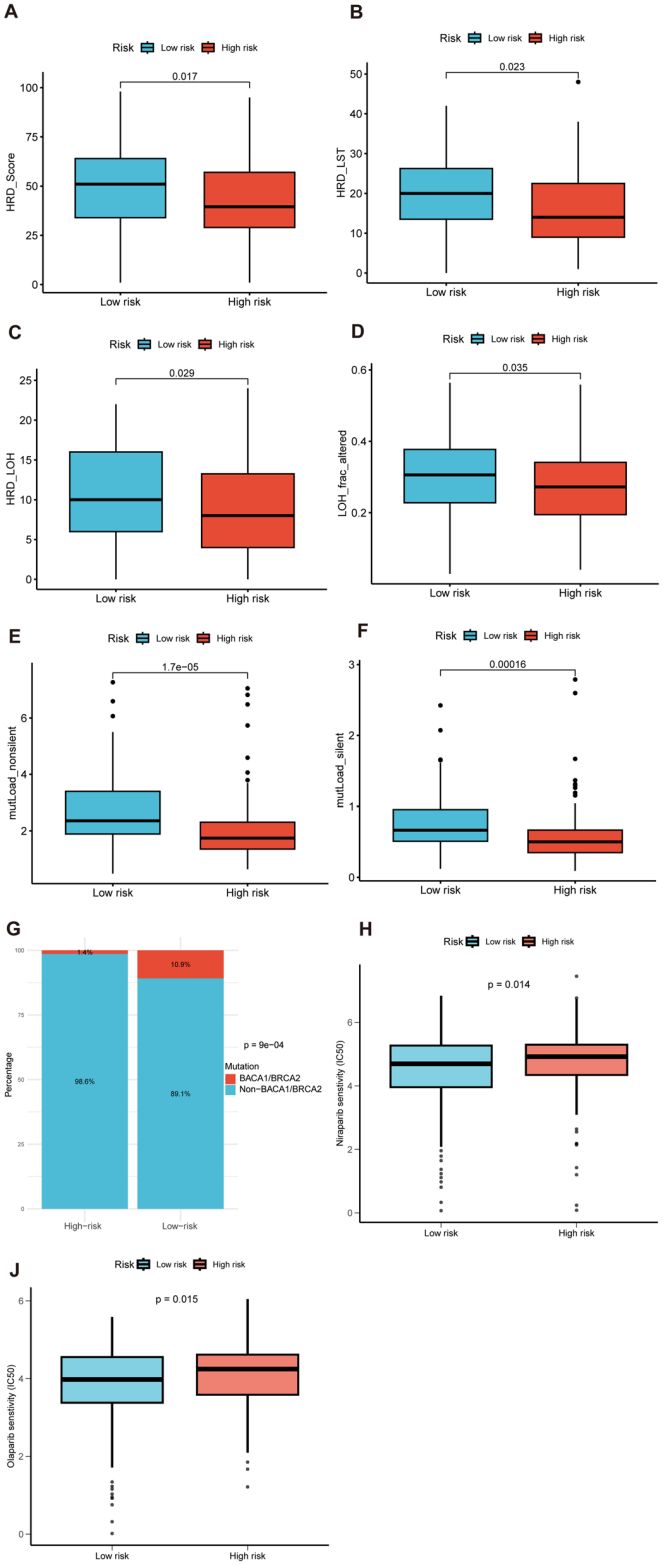
Investigation of HRD in the high-risk and low-risk subgroups. **A**–**F** Comparison of HRD_scores, HRD_LST, HRD_LOH, LOH_frac_altered, mutLoad_nosilent, and mutLoad_silent between the low-risk and high-risk groups. **G** The different percentages of BRAC1/2 mutation between the risk groups. **H**–**J** The different IC50 for PARPi drugs Niraparib and Olaparib

Considering the crucial role of HRD and PARPi in OC treatment, we investigated the relationship between the risk score and HRD as well as PARPi. Our findings revealed that the HRD_score, HRD_LST, HRD_LOH, and LOH_frac_altered were higher in the low-risk score group (Fig. 12A–D). Moreover, the mutLoad_nosilent and mutLoad_silent were also higher in the low-risk score group (Fig. 12E, F). Furthermore, we examined the distribution of BRCA1/2 gene mutations in the high-risk and low-risk subgroups using mutation data. It was evident that the percentage of patients with mutations was significantly higher in the low-risk group (Fig. 12G). In addition, the low-risk group exhibited lower IC50 for PARPi drugs, such as Niaparib and Olaparib (Fig. 12H, J). These results suggested at patients in the low-risk group may be more sensitive to PARPi treatment.

### Compared with previous risk models

**Fig. 13:**
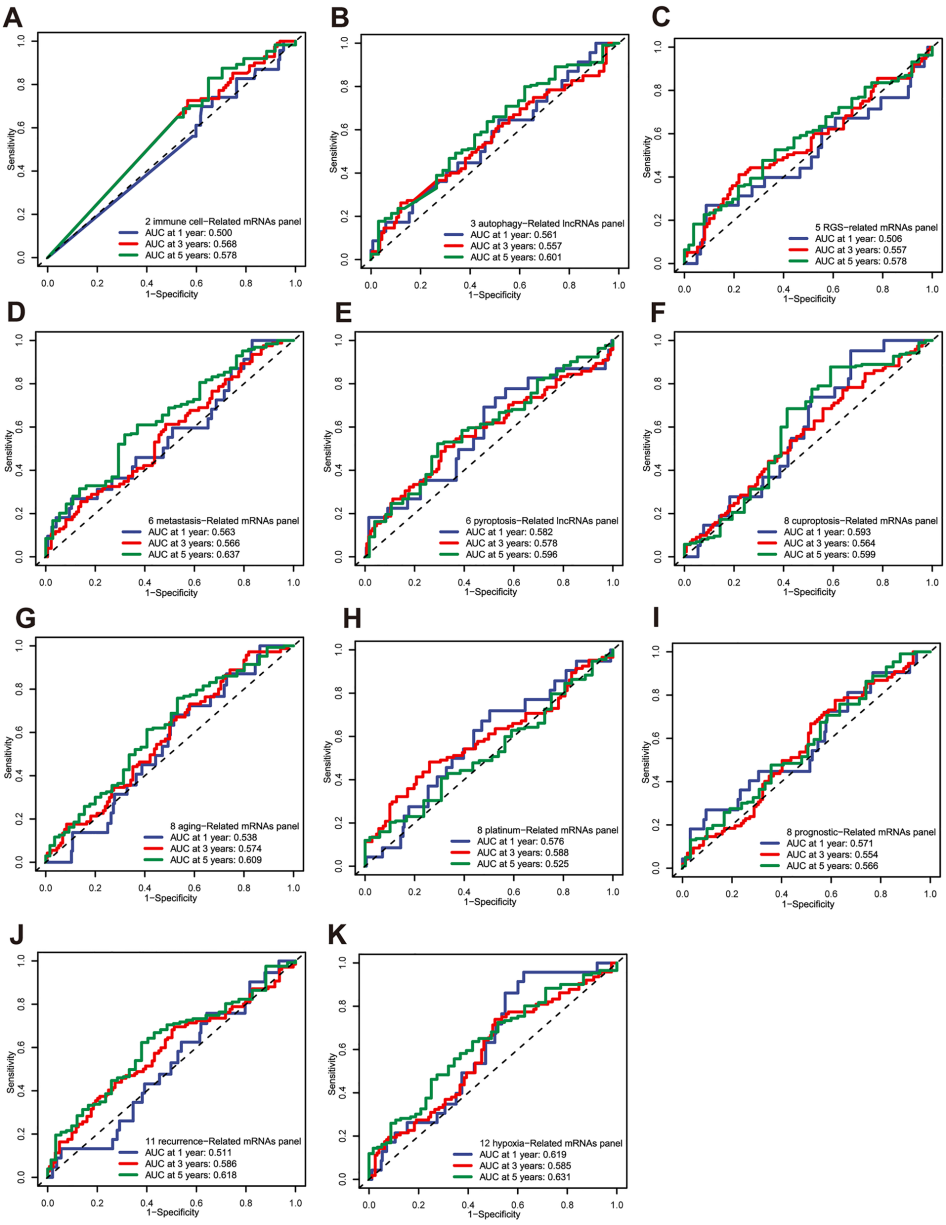
ROC compared with the previous models. **A**–**K** ROC curves were generated to evaluate the predictive accuracy of the risk score at 1, 3, and 5 years in the previously published studies

We reviewed the literature on previously published prognostic models in OC and compared the ROC curves with other established risk models. This comparison included a panel of two mRNAs signature (CXCL13, IL26) [27] (Fig. 13A), a panel of three lncRNAs (AC136601.1, LINC02273, AC011445.1) [28] (Fig. 13B), a panel of 5 RGS-related mRNAs (RGS11, RGS10, RGS13, RGS4, RGS3) [29] (Fig. 13C), a panel of 6 metastasis-related mRNAs (TIMP3, FBN1, IGKC, RPL21, UCHL1, RARRES1) [30] (Fig. 13D), a panel of 6 pyroptosis-related lncRNAs (AC006001.2, LINC02585, AL136162.1, AC005041.3, AL023583.1, LINC02881) [31] (Fig. 13E), a panel of 8 cuproptosis-related mRNAs (AMER1, ATP2A3, HIPK2, RRP12, VANGL1, JAG2, GALNT6, CD79A) [32] (Fig. 13F), a panel of 8 aging-related mRNAs (JAK2, IL2RG, EEF1E1, UBB, EPS8, FOXO1, STAT5A, PAPPA) [33] (Fig. 13G), a panel of 8 platinum-related mRNAs (GJA8, PNLDC1, SLC5A1, VSTM2L, CACNA1C, SEZ6L, GDF3, SYNM) [34] (Fig. 13H), a panel of 8 prognostic-related mRNAs (ACTN3, ESRRB, DCN, PSMC4, CXCR4, FBP1, ARTN, GMPPB) [35] (Fig. 13I), a panel of 11 recurrence-related mRNAs (BIRC3, CDH2, CDH6, DDIT4, GAS1, IFIT1, IGF2, ISLR, MUC16, SAD2, DIRAS3) [36] (Fig. 13J), a panel of 12 hypoxia-related mRNAs (CLDN4, EPCAM, MCM3, CXCL13, MIF, FOXO1, UBB, SEC22B, TCEAL4, ECI2, OGN, CFI) [37] (Fig. 13K). The detailed expression of risk genes, along with the corresponding risk scores and risk group assignments, could be found in Additional file 2: Table S7. It was observed that the predictive performance of our signature surpassed that of all the aforementioned risk models.

### Identification of the two distinct subtypes of OC

**Fig. 14:**
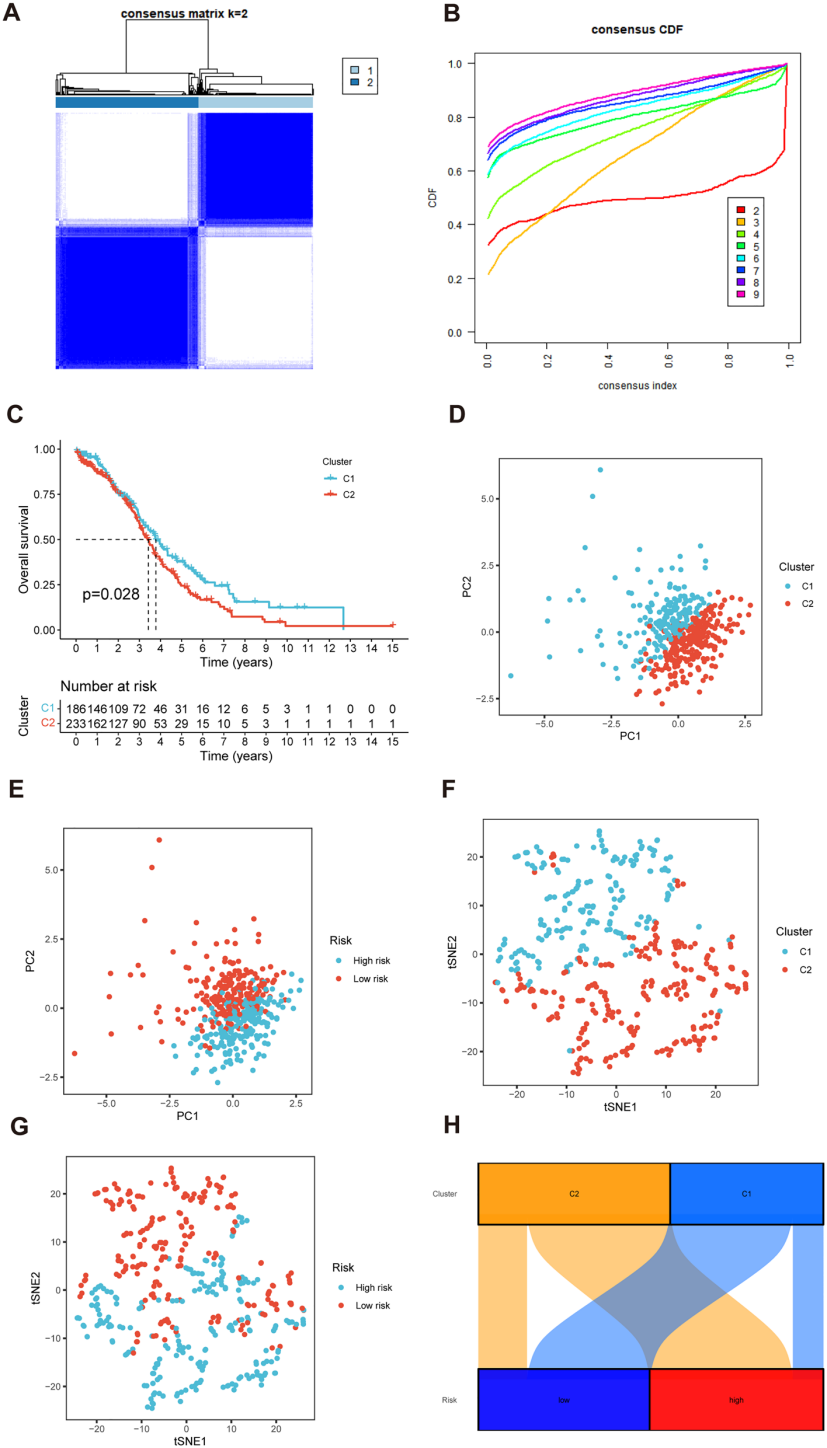
Two distinct expression clusters characterized by consensus clustering analysis. **A**, **B** Patients were divided into three clusters by ConsensusClusterPlus. **C** Kaplan–Meier survival curves of OS in two clusters. **D**, **E** PCA of risk groups and clusters. **F**, **G** t-SNE of risk groups and clusters. **H** Sankey diagram of clusters with their risk types and survival status

We used consensus clustering based on the 4 CD8TEXGs expression which came from the risk model; two distinct clusters were displayed (Fig. 14A, B). Survival analysis demonstrated a significant difference between the two clusters (Fig. 14C). Principal component analysis (PCA) and t-SNE (t-distributed Stochastic Neighbor Embedding) analysis of 4 CD8TEXGs expression was divided into two clusters, and the pre-defined high- and low-risk groups could also be divided into two clusters (Fig. 14D–G), and the Sankey diagram was adopted to display relationships of clusters with their risk types, clusters, and survival status (Fig. 14H).

### Risk gene expression in cell lines

**Fig. 15:**
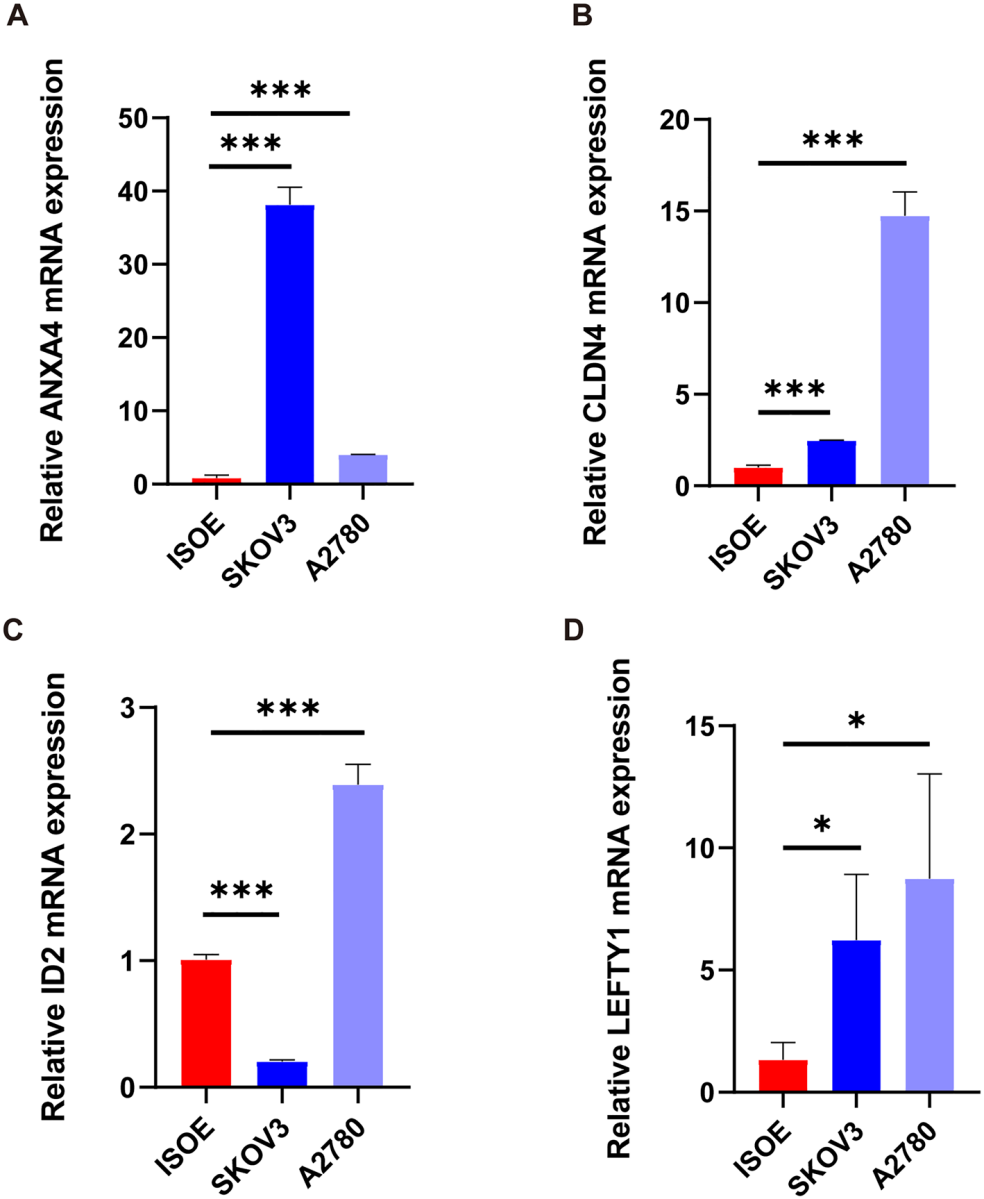
Risk gene expression in cell lines. **A**–**D** Real-time PCR to quantify the expression level of risk genes (ANXA4, CLDN4, ID2, and LEFTY1) expression levels in three OC cell lines. ISOE, SKOV3 and A2780

We used real-time PCR to quantify the expression level of risk genes in three OC cell lines (ISOE, SKOV3, and A2780). ANXA4, CLDN4, ID2, LEFTY1 expression were significant different in the cell lines (Fig. 15A–D).

## Discussion

OC was the primary cause of mortality among gynecologic malignancies globally, exhibiting a high mortality-to-incidence ratio and accounting for the largest proportion of gynecologic cancers. While many patients achieved a complete response following primary treatment involving surgical resection and chemotherapy, a significant proportion (65–80%) experience recurrence within the first five years with resistance to chemotherapy [1, 38, 39]. Over the past two decades, there has been accumulating evidence supporting the widespread use of immunotherapies in the clinical treatment of various tumor types. Despite the advancements in immune modulators (e.g., checkpoint inhibitors and cytokines), targeted antibodies (e.g., monoclonal antibodies), and adoptive cell therapy (e.g., chimeric antigen receptor (CAR)- and TCR-engineered T cells), the response rates to immunotherapy among OC patients have remained modest. Therefore, there was a pressing need to explore additional biomarkers that may aid non-responsive patients. The combination of therapeutic immunotherapy and chemotherapeutic approaches holds great potential in significantly improving treatment efficiency.

CD8+T lymphocytes constituted a specialized population of T cells and played a crucial role in adaptive cytotoxic T cell responses against chronic infections and cancer [40, 41]. However, impaired clearance of chronic viral infections and tumors has been attributed to CD8+T cell exhaustion, which was a differentiation state characterized by reduced and altered effector function. This exhaustion can be partially reversed by blocking inhibitory receptors [42]. Recent advancements in technology, particularly the rapid development of single-cell omics and pathomics, have significantly contributed to our understanding of T cell exhaustion. These advancements have revealed the existence of distinct subsets of exhausted CD8+T cells with varying transcriptional and epigenetic profiles, functional states, and responses to therapeutic interventions. CD8+T cell exhaustion was a common phenomenon in cancer and chronic viral infections could be reversed [43–46], but the effectiveness of existing therapies was not universally applicable or durable. Currently, we lacked the ability to predict which patients would respond to these therapies, and the mechanisms underlying treatment success or failure remain poorly understood [47, 48].

In the field of precision genomic medicine, numerous predictive signatures have been developed to improve our understanding of patient prognosis outcomes across various cancer types. These signatures rely on the analyses of single-cell and bulk transcriptome data, enabling a more precise approach to genomic medicine. Such as fibroblasts related risk signature in bladder urothelial carcinoma [49], an early monocyte gene signature in acute respiratory distress syndrome [50], a cuproptosis-related genes signature in hepatocellular carcinoma [51], an NK cell marker genes signature in lung adenocarcinoma [52], a B cell marker genes signature in clear cell renal cell carcinoma [53]. However, there were no known studies with CD8+T cells related signatures, such as exhausted CD8+T cells-related genes signature in OC. The recent utilization of scRNA-seq has provided valuable insights into the tumor microenvironment (TME). This technology has facilitated a comprehensive understanding of the biological characteristics and heterogeneity of tumor-infiltrating immune cells. Furthermore, it has shed light on their potential roles in tumor progression and their responsiveness to immune checkpoint inhibitors and other immunotherapies. In the present study, we developed a novel risk signature for predicting prognosis and survival in OC. This signature was constructed based on the genes associated with exhausted CD8+T cells, utilizing both scRNA-seq and TCGA bulk sequencing datasets. We first performed internal validation by splitting the TCGA bulk sequencing datasets into train and test subsets at a 1:1 ratio. Subsequently, we validated the prognostic value of the risk signature for OS, PFS and DFS using GEO datasets. Our results demonstrated the robustness of the risk signature. Furthermore, multivariate Cox regression analysis confirmed the risk signature as an independent prognostic factor in multiple GEO datasets. To enhance its clinical applicability, we developed a nomogram integrating the risk score. The accuracy of the risk signature was assessed through calibration curves and ROC analysis, yielding promising results. Additionally, we compared our model with previously published risk models in OC and found our model to be superior. We observed that patients in the low-risk group exhibited higher HRD scores and a higher prevalence of BRCA1/2 mutations. Consistently, the low-risk group showed increased sensitivity to PARPi. Moreover, the low-risk group displayed higher TMB, suggesting a potential suitability for immunotherapy. Furthermore, when combined with TMB and other clinical information, the risk score proved to be an independent predictor. We also found that the low-risk group had higher expression of PD-1 and PD-L1. The elevated expression of PD-L1 in the low-risk subgroup may seem inconsistent with traditional knowledge, after extensive literature review, we found that this phenomenon has been reported in various cancer types and is not an isolated case. Yi et al. indicated that patients with low-risk scores had modestly increased PD-L1 and significantly elevated PD-1 and CTLA-4 expressions [54], Liu et al. reported that that the expression levels of PD-1, PD-L1 were remarkably higher in the low-risk groups [55], Kairaet et al. showed that stromal CD4 tumor-infiltrating lymphocytes (TILs) were identified as a significant marker for predicting the PFS after pembrolizumab therapy and especially in patients with non-adenocarcinoma and high PD-L1 expression [56], Li et al. elucidated that higher expressions of PD-1 and PD-L1 correlates with better prognosis of CRC patients and TILs-PD-1 is an independent prognostic factor for OS and DFS of CRC patients, especially for MMR-proficient subgroup [57], Beckers et al. observed that cytoplasmic, stromal PD-L1 expression were both associated with a good outcome in this cohort and cytoplasmic expression of PD-L1 ≥ 5% was associated with improved patient survival for breast cancer-specific deaths [58], Zhu revealed that patients expressing PD-L1 (positive PD-L1 expression) had a longer median PFS and a longer median OS compared with those not expressing PD-L1 (negative) [59], Bae demonstrated that PD-L1 expression was significantly associated with better DFS and OS [60]. Based on this analysis, we proposed that the high expression of PD-L1 in the low-risk subgroup may reflect a more active anti-tumor immune state rather than simple immune suppression. This state may be associated with higher levels of TILs, stronger anti-tumor immune responses, and potentially better responses to immunotherapy. In functional enrichment analysis, pathways associated with tumor metabolism were found to be activated in the high-risk group. Additionally, the four identified CD8TEXGs showed close associations with cancer and cell development. Lin et al. indicated that CLDN4 regulated the Epithelial–Mesenchymal Transition (EMT) in OC [61]. Gao et al. demonstrated that C-Terminus of clostridium perfringens enterotoxin downregulates CLDN4 and sensitized OC Cells to taxol and carboplatin [62]. Kwon et al. showed that derepression of CLDN4 during ovarian tumorigenesis is associated with loss of repressive histone modifications [63]. Shang et al. revealed that regulated sensitivity to cisplatin by controlling expression of the copper and cisplatin influx transporter CTR1 [64]. Kuang evidenced that ELF3 suppresses miR-485-5p transcription to enhance CLDN4 expression, leading to Wnt/β-catenin activation and promoting OC cell growth and metastasis [65]. Loss of e-cadherin led to ID2-dependent inhibition of cell cycle progression in metastatic lobular breast cancer [66]. ID2 inhibited innate antiviral immunity by blocking TBK1- and IKKε-induced activation of IRF3 [67]. ID2 and HIF-1α collaborated to protect quiescent hematopoietic stem cells from activation, differentiation, and exhaustion [68]. Liu et al. demonstrated that wild p53 activates ANXA4 transcription, promotes its expression and enhances NF‑κB p50 and ANXA4 interaction. This in turn activates the NF‑κB signaling pathway, promotes cell cycle progression and inhibits apoptosis, thus contributing to the malignant progression of OC [69]. Toyama et al. proposed that ANXA4 was candidate subtype-specific biomarkers that could help define the basis of tumor histology at a molecular level by proteomic [70]. Mogami et al. demonstrated that ANXA4 was involved in proliferation, chemo-resistance and migration and invasion in OC [71]. Matsumoto et al. proposed that TGF-β-mediated LEFTY1/Akt/GSK-3β/Snail axis modulates EMT and cancer stem cell properties in OC [72]. Akiya et al. demonstrated that blocking LEFTY1 expression with a specific short hairpin RNA inhibited cisplatin-induced apoptosis, probably through the increased expression of both XIAP and bcl2, but not bax in OC [73]. We further assessed the gene expression of the risk model using quantitative real-time PCR. The above results showed the novelty and reliability of our risk model. Compared to the previous studies, the innovative aspects of this study were reflected in the following points: (1) This was the first study to systematically investigate the role of CD8+T cell exhaustion in OC prognosis; (2) We identified OC-specific exhausted CD8+T cells-related genes using scRNA-seq data and validated them in large sample cohorts; (3) The constructed risk score model could not only predict OS, PFS, DFS, but also was closely related to HRD, TMB, and PARPi sensitivity, showing broad clinical application prospects; (4) Our study provided new clues and directions for the role of CD8+T cell exhaustion in OC immunotherapy. In summary, this study deepened our understanding of the immune microenvironment in OC and laid the foundation for future research. However, this study did have limitations. Firstly, our findings required prospective validation through multicenter study cohorts to strengthen their validity. Secondly, further investigations were needed to explore the functions and molecular mechanisms of the identified four CD8TEXGs in OC, employing additional in vitro and in vivo experiments. Nonetheless, our study provided valuable insights into the identification of CD8TEXGs as potential prognostic biomarkers and therapeutic targets, offering promising clinical predictive value.

## Conclusion

In conclusion, we identified four CD8TEXGs incorporated into a risk model as biomarkers in OC, utilizing scRNA-seq datasets, TCGA bulk-seq datasets, and GEO datasets. Notably, significant differences in survival rate, HRD status, and TMB status were observed between the high-risk and low-risk groups, indicating the potential of these biomarkers to predict clinical outcomes and potentially serve as therapeutic targets for OC patients. As our understanding of cancer immunotherapy continues to evolve, our study provided novel insights into the role of CD8TEXGs in the treatment of OC.

### Supplementary Information

Additional file 1: Table S1. Four risk genes primer sequences. Table S2. Cell markers of cell subsets. Table S3. The list of exhausted CD8+T cells-related genes. Table S4. The genes list after performing univariate Cox regression analysis. Table S5. Clinical features comparison between high-risk and low-risk subgroups. Table S6. The concrete clinical information for TCGA whole dataset patients.

Additional file 2: Table S7. The detailed risk genes expression, risk score, and risk group in all models.

## Data Availability

Not applicable.
