# Supplementary material for: Establishment of an ovarian cancer exhausted CD8+T cells-related genes model by integrated analysis of scRNA-seq and bulk RNA-seq
Source: Eur J Med Res. 2024 Jul 5;29:358. doi: 10.1186/s40001-024-01948-8 (PMC11225302; doi:10.1186/s40001-024-01948-8)
Supplement: Supplementary file 1 — Additional file 1: Table S1. Four risk genes primer sequences. Table S2. Cell markers of cell subsets. Table S3. The list of exhausted CD8+T cells-related genes. Table S4. The genes list after performing univariate Cox regression analysis. Table S5. Clinical features comparison between high-risk and low-risk subgroups. Table S6. The concrete clinical information for TCGA whole dataset patients. [file 40001_2024_1948_MOESM1_ESM.docx]

| gene | name | sequence |
| --- | --- | --- |
| ANXA4 | ANXA4-F | GGAGGTACTGTCAAAGCTGCT |
|  | ANXA4-R | GGCAAGGACGCTAATAATGGC |
| CLDN4 | CLDN4-F | GGGGCAAGTGTACCAACTG |
|  | CLDN4-R | GACACCGGCACTATCACCA |
| ID2 | ID2-F | AGTCCCGTGAGGTCCGTTAG |
|  | ID2-R | AGTCGTTCATGTTGTATAGCAGG |
| LEFTY1 | LEFTY1-F | CCGCGGAAAGAGGTTCAGCCA |
|  | LEFTY1-R | GCTGCTCCATGCCGAACACCA |

Additional file 1: Table S1. Four risk genes primer sequences

Additional file 1: Table S2. Cell markers of cell subsets.

| Cell types | Markers |  |  |  |  |
| --- | --- | --- | --- | --- | --- |
| CD8Tex | CD3D | CTLA4 | TIGIT | GZMA | CD8A |
| Malignant | KRT19 | KRT18 | LCN2 | EPCAM |  |
| Mono/Macro | C1QA | CD14 | HLA-DQB1 |  |  |
| Myofibroblasts | ACTA2 | RGS5 | MCAM |  |  |
| Fibroblasts | DCN | LUM | FAP | COL3A1 |  |

Additional file 1: Table S3. The list of exhausted CD8+ T cell-related genes.

| gene |
| --- |
| RGS1 |
| CD2 |
| CD52 |
| SRGN |
| PTPRC |
| BATF |
| CD3D |
| CTLA4 |
| SAMSN1 |
| IL2RG |
| DOK2 |
| RAC2 |
| RHOH |
| LTB |
| SLA |
| IL7R |
| TNFRSF4 |
| CD48 |
| CD69 |
| PTPRCAP |
| CD96 |
| CD247 |
| CD53 |
| GNLY |
| ARHGDIB |
| B2M |
| IFNG |
| LCP1 |
| XCL1 |
| GZMA |
| CORO1A |
| GZMB |
| GMFG |
| HLA-B |
| BTG1 |
| TNFRSF1B |
| HLA-A |
| TNFSF14 |
| ITM2A |
| EPCAM |
| TSC22D3 |
| CLDN4 |
| CLEC2D |
| TTN |
| SLPI |
| TBC1D4 |
| HLA-C |
| IL32 |
| GATA3 |
| CXCR4 |
| KRT18 |
| ISG20 |
| DNAJB1 |
| HLA-F |
| CREM |
| CYTIP |
| SPINT2 |
| ELF3 |
| RGS2 |
| MDK |
| GSTP1 |
| CLDN3 |
| WFDC2 |
| KRT8 |
| DSP |
| CCND2 |
| HSPA5 |
| CP |
| KRT19 |
| PTTG1IP |
| S100A4 |
| PIM2 |
| MAF |
| PAX8 |
| DUSP4 |
| CALD1 |
| XAF1 |
| ID2 |
| PERP |
| MT1E |
| TM4SF1 |
| CACYBP |
| KRT7 |
| HES1 |
| IGFBP7 |
| CLEC2B |
| TPM1 |
| TFPI2 |
| EHF |
| SCGB2A1 |
| RBP1 |
| FOLR1 |
| TSPAN1 |
| TACSTD2 |
| ASRGL1 |
| LAMB1 |
| ID4 |
| SERPING1 |
| CRYAB |
| CFB |
| ANKRD12 |
| ADAMTS1 |
| ANXA4 |
| CXCL2 |
| LGALS3 |
| SH3BGRL3 |
| LEFTY1 |
| STK17B |
| SP110 |
| NMU |
| TSPYL2 |
| GPX3 |
| TIMP1 |
| ELF1 |
| CLU |
| VCAN |
| ZNF331 |
| COL4A1 |
| CXCL1 |
| CENPF |
| SPARC |
| REL |
| COL1A2 |
| SPP1 |
| FN1 |
| NTS |
| COL3A1 |
| COL1A1 |
| DCN |
| FKBP11 |
| SPARCL1 |
| RGS5 |

Additional file 1: Table S4. The genes list after performing univariate Cox regression analysis.

| gene |
| --- |
| CLDN4 |
| ELF3 |
| ID2 |
| TFPI2 |
| TACSTD2 |
| CRYAB |
| ANXA4 |
| LEFTY1 |

Additional file 1: Table S5. Clinical features comparison between high-risk and low-risk subgroups.

| Covariates | Type | Total | Test | Train | Pvalue |
| --- | --- | --- | --- | --- | --- |
| age | <=50 | 101(24.11%) | 51(24.4%) | 50(23.81%) | 0.978 |
| age | >50 | 318(75.89%) | 158(75.6%) | 160(76.19%) |  |
| grade | G1 | 1(0.24%) | 1(0.48%) | 0(0%) | 0.4569 |
| grade | G2 | 47(11.22%) | 26(12.44%) | 21(10%) |  |
| grade | G3 | 360(85.92%) | 178(85.17%) | 182(86.67%) |  |
| grade | unknow | 11(2.63%) | 4(1.91%) | 7(3.33%) |  |
| stage | Stage I | 1(0.24%) | 0(0%) | 1(0.48%) | 0.6382 |
| stage | Stage II | 24(5.73%) | 14(6.7%) | 10(4.76%) |  |
| stage | Stage III | 327(78.04%) | 162(77.51%) | 165(78.57%) |  |
| stage | Stage IV | 64(15.27%) | 32(15.31%) | 32(15.24%) |  |
| stage | unknow | 3(0.72%) | 1(0.48%) | 2(0.95%) |  |
| tumor residual | R0 | 75(17.9%) | 31(14.83%) | 44(20.95%) | 0.2162 |
| tumor residual | R1 | 195(46.54%) | 101(48.33%) | 94(44.76%) |  |
| tumor residual | R2 | 28(6.68%) | 16(7.66%) | 12(5.71%) |  |
| tumor residual | unknow | 121(28.88%) | 61(29.19%) | 60(28.57%) |  |

Additional file 1: Table S6. The concrete clinical information for TCGA whole dataset patients.

| Sample age grade stage tumor residual |
| --- |
| TCGA-09-1672 78 G1 Stage III unknow |
| TCGA-61-1721 38 G1 Stage IV unknow |
| TCGA-36-2540 26 G1 Stage III unknow |
| TCGA-61-2017 64 G1 Stage III R1 |
| TCGA-10-0925 58 G1 Stage III R1 |
| TCGA-09-1664 37 G1 Stage III R1 |
| TCGA-04-1335 60 G2 Stage I unknow |
| TCGA-04-1337 78 G2 Stage III R0 |
| TCGA-31-1959 49 G2 Stage IV unknow |
| TCGA-61-2003 53 G2 Stage III R1 |
| TCGA-30-1862 65 G2 Stage IV unknow |
| TCGA-42-2590 57 G2 Stage IV unknow |
| TCGA-29-2436 36 G2 Stage III R1 |
| TCGA-29-1777 47 G2 Stage III unknow |
| TCGA-29-1775 51 G2 Stage III R1 |
| TCGA-61-1916 53 G2 Stage III R1 |
| TCGA-29-1705 47 G2 Stage III unknow |
| TCGA-04-1342 80 G2 Stage IV unknow |
| TCGA-31-1950 76 G2 Stage III R1 |
| TCGA-13-1485 48 G2 Stage IV R1 |
| TCGA-04-1369 53 G2 Stage III R1 |
| TCGA-29-1771 76 G2 Stage III unknow |
| TCGA-30-1887 67 G2 Stage III R1 |
| TCGA-29-1770 54 G2 Stage III R1 |
| TCGA-36-1577 43 G2 Stage II R0 |
| TCGA-04-1648 57 G2 Stage III R1 |
| TCGA-30-1891 61 G2 Stage III R1 |
| TCGA-61-2008 40 G2 Stage II R0 |
| TCGA-61-2012 81 G2 Stage II unknow |
| TCGA-25-2408 37 G2 Stage IV R1 |
| TCGA-29-1710 54 G2 Stage III R1 |
| TCGA-04-1652 76 G2 Stage III unknow |
| TCGA-29-1696 43 G2 Stage III R1 |
| TCGA-29-1711 45 G2 Stage III R0 |
| TCGA-09-2045 50 G2 Stage IV R1 |
| TCGA-30-1866 61 G2 Stage IV R1 |
| TCGA-61-1919 58 G2 Stage III unknow |
| TCGA-29-1766 74 G2 Stage III R2 |
| TCGA-29-1695 62 G2 Stage III unknow |
| TCGA-09-1665 73 G2 Stage III R0 |
| TCGA-29-2431 59 G2 Stage III R1 |
| TCGA-61-1743 53 G2 Stage II R0 |
| TCGA-59-2372 73 G2 Stage III R0 |
| TCGA-04-1655 49 G2 Stage III R0 |
| TCGA-29-1690 66 G2 Stage III R2 |
| TCGA-04-1654 69 G2 Stage III R1 |
| TCGA-29-1691 51 G2 Stage III R1 |
| TCGA-13-1495 60 G2 Stage III R1 |
| TCGA-13-1477 49 G2 Stage IV R1 |
| TCGA-61-2101 55 G2 Stage III R2 |
| TCGA-04-1514 45 G2 Stage III R0 |
| TCGA-61-2096 56 G2 Stage I R0 |
| TCGA-29-1703 56 G2 Stage III R1 |
| TCGA-61-2097 71 G2 Stage II R1 |
| TCGA-61-2095 54 G2 Stage III R0 |
| TCGA-13-1482 52 G2 Stage IV unknow |
| TCGA-09-1667 61 G2 Stage II R0 |
| TCGA-29-1764 49 G2 Stage III unknow |
| TCGA-29-2425 60 G2 Stage III unknow |
| TCGA-04-1351 76 G2 Stage III unknow |
| TCGA-61-2098 62 G2 Stage III R2 |
| TCGA-04-1346 73 G2 Stage III unknow |
| TCGA-29-1763 43 G2 Stage II R1 |
| TCGA-09-2050 65 G2 Stage II R0 |
| TCGA-20-0996 59 G2 Stage III R1 |
| TCGA-61-2104 53 G2 Stage II R0 |
| TCGA-29-1688 39 G2 Stage III unknow |
| TCGA-10-0927 65 G2 Stage III R1 |
| TCGA-13-1489 70 G2 Stage III unknow |
| TCGA-04-1542 52 G2 Stage III R1 |
| TCGA-29-2414 75 G2 Stage III R2 |
| TCGA-29-1762 59 G2 Stage IV unknow |
| TCGA-13-1481 76 G2 Stage III R1 |
| TCGA-29-1692 58 G2 Stage III R2 |
| TCGA-09-1675 50 G2 Stage I R0 |
| TCGA-30-1857 64 G3 Stage IV unknow |
| TCGA-23-1107 59 G3 Stage IV unknow |
| TCGA-25-1631 73 G3 Stage III R1 |
| TCGA-24-2262 57 G3 Stage III R1 |
| TCGA-23-1809 63 G3 Stage II R1 |
| TCGA-61-1722 89 G3 Stage I R0 |
| TCGA-24-1422 82 G3 Stage III R1 |
| TCGA-04-1519 48 G3 Stage III unknow |
| TCGA-24-2261 76 G3 Stage III R2 |
| TCGA-24-2288 70 G3 Stage III unknow |
| TCGA-25-1312 69 G3 Stage IV R1 |
| TCGA-25-2392 75 G3 Stage IV R2 |
| TCGA-04-1341 85 G3 unknow unknow |
| TCGA-29-2435 78 G3 Stage IV R2 |
| TCGA-61-2016 51 G3 Stage III R0 |
| TCGA-24-1471 60 G3 Stage III unknow |
| TCGA-61-1895 52 G3 Stage III R0 |
| TCGA-42-2593 67 G3 Stage III unknow |
| TCGA-57-1585 57 G3 Stage III R2 |
| TCGA-61-1995 43 G3 Stage III R1 |
| TCGA-25-1317 66 G3 Stage III unknow |
| TCGA-59-2355 58 G3 Stage IV unknow |
| TCGA-36-2543 85 G3 Stage III unknow |
| TCGA-61-1740 71 G3 Stage III unknow |
| TCGA-30-1855 61 G3 Stage III R1 |
| TCGA-13-0755 75 G3 Stage IV R1 |
| TCGA-13-1510 62 G3 Stage III R0 |
| TCGA-20-1687 46 G3 Stage IV R0 |
| TCGA-13-0724 72 G3 Stage IV R1 |
| TCGA-23-1032 73 G3 Stage IV R1 |
| TCGA-23-2641 85 G3 Stage III R1 |
| TCGA-20-1686 75 G3 Stage III R0 |
| TCGA-25-2401 64 G3 Stage III R1 |
| TCGA-25-1322 62 G3 Stage IV unknow |
| TCGA-09-1673 50 G3 Stage IV unknow |
| TCGA-25-2396 71 G3 Stage III R0 |
| TCGA-13-1512 49 G3 Stage III R0 |
| TCGA-23-1111 63 G3 Stage III unknow |
| TCGA-24-1470 54 G3 Stage III R1 |
| TCGA-24-1843 66 G3 Stage III R1 |
| TCGA-24-1844 64 G3 Stage III R1 |
| TCGA-24-1845 42 G3 Stage III R1 |
| TCGA-23-2649 60 G3 Stage III R1 |
| TCGA-13-1511 52 G3 Stage IV unknow |
| TCGA-13-1509 64 G3 Stage IV R1 |
| TCGA-13-1505 63 G3 Stage III R1 |
| TCGA-13-1411 81 G3 Stage III unknow |
| TCGA-13-1496 65 G3 Stage III unknow |
| TCGA-23-1120 60 G3 Stage III R1 |
| TCGA-61-1730 78 G3 Stage I R1 |
| TCGA-24-1846 45 G3 Stage III R1 |
| TCGA-13-1506 45 G3 Stage III unknow |
| TCGA-23-2647 49 G3 Stage III R1 |
| TCGA-23-2645 54 G3 Stage III R0 |
| TCGA-09-2048 63 G3 Stage III R1 |
| TCGA-23-1116 83 G3 Stage III unknow |
| TCGA-13-0921 72 G3 Stage III R0 |
| TCGA-23-2643 74 G3 Stage III R1 |
| TCGA-61-2088 51 G3 Stage III R0 |
| TCGA-13-1507 77 G3 Stage III R1 |
| TCGA-61-2087 49 G3 Stage I R0 |
| TCGA-24-1427 58 G3 Stage III unknow |
| TCGA-24-2019 46 G3 Stage III R1 |
| TCGA-13-1412 41 G3 Stage IV R1 |
| TCGA-24-1426 43 G3 Stage III R1 |
| TCGA-29-1784 55 G3 Stage III R0 |
| TCGA-59-2363 40 G3 Stage III R0 |
| TCGA-61-1998 48 G3 Stage III R1 |
| TCGA-24-1850 72 G3 Stage III R1 |
| TCGA-61-1900 51 G3 Stage III R0 |
| TCGA-24-1849 80 G3 Stage III R1 |
| TCGA-13-1408 59 G3 Stage III unknow |
| TCGA-24-1425 45 G3 Stage III R1 |
| TCGA-24-1424 67 G3 Stage III R1 |
| TCGA-13-1407 51 G3 Stage III unknow |
| TCGA-36-2549 54 G3 Stage II R0 |
| TCGA-13-0924 45 G3 Stage IV R1 |
| TCGA-09-2044 77 G3 Stage II R0 |
| TCGA-09-2043 69 G3 Stage I R0 |
| TCGA-13-0714 55 G3 Stage IV R1 |
| TCGA-24-1423 61 G3 Stage III R1 |
| TCGA-13-2071 63 G3 Stage IV R1 |
| TCGA-24-1413 51 G3 Stage III R1 |
| TCGA-61-2612 63 G3 Stage III R1 |
| TCGA-24-1416 34 G3 Stage IV R1 |
| TCGA-23-1121 51 G3 Stage III unknow |
| TCGA-13-0923 74 G3 Stage III R0 |
| TCGA-61-2102 74 G3 Stage III unknow |
| TCGA-13-1404 48 G3 Stage III R1 |
| TCGA-10-0934 50 G3 Stage III R1 |
| TCGA-31-1953 52 G3 Stage III unknow |
| TCGA-13-1409 73 G3 Stage III R1 |
| TCGA-29-1783 58 G3 Stage III R1 |
| TCGA-13-1405 49 G3 Stage IV R1 |
| TCGA-42-2589 54 G3 Stage III R1 |
| TCGA-24-0966 78 G3 Stage III unknow |
| TCGA-24-0980 53 G3 Stage III unknow |
| TCGA-13-0799 44 G3 Stage III R0 |
| TCGA-24-1417 54 G3 Stage IV R1 |
| TCGA-24-1419 62 G3 Stage III R2 |
| TCGA-24-1418 68 G3 Stage III unknow |
| TCGA-13-0800 52 G3 Stage III R0 |
| TCGA-13-0802 79 G3 Stage III R0 |
| TCGA-13-0801 46 G3 Stage III R0 |
| TCGA-24-1842 49 G3 Stage III R1 |
| TCGA-29-1781 69 G3 Stage III R0 |
| TCGA-13-A5FU 60 G3 Stage IV R1 |
| TCGA-61-1899 81 G3 Stage III R0 |
| TCGA-13-0768 73 G3 Stage III R1 |
| TCGA-24-1436 57 G3 Stage III R1 |
| TCGA-09-1674 79 G3 Stage III R1 |
| TCGA-36-1575 83 G3 Stage III R0 |
| TCGA-13-2060 51 G3 Stage IV R1 |
| TCGA-61-2614 71 G3 Stage III R1 |
| TCGA-13-0797 49 G3 Stage III R1 |
| TCGA-13-2065 71 G3 Stage III R1 |
| TCGA-23-1029 46 G3 Stage III R1 |
| TCGA-61-1904 60 G3 Stage III R1 |
| TCGA-42-2587 75 G3 Stage III unknow |
| TCGA-24-1469 71 G3 Stage III R1 |
| TCGA-59-2352 78 G3 Stage III unknow |
| TCGA-42-2588 61 G3 Stage IV unknow |
| TCGA-09-0365 70 G3 Stage III R0 |
| TCGA-13-2066 49 G3 Stage IV R1 |
| TCGA-24-1920 74 G3 Stage III R1 |
| TCGA-09-1659 51 G3 Stage III unknow |
| TCGA-WR-A838 72 G3 Stage III unknow |
| TCGA-24-1565 74 G3 Stage III R1 |
| TCGA-13-2061 58 G3 Stage III R0 |
| TCGA-13-1403 48 G3 Stage III unknow |
| TCGA-24-1928 77 G3 Stage III R2 |
| TCGA-13-0757 71 G3 Stage III R1 |
| TCGA-24-1847 45 G3 Stage IV R1 |
| TCGA-57-1583 57 G3 Stage III R0 |
| TCGA-13-0758 60 G3 Stage IV R1 |
| TCGA-61-1901 65 G3 Stage IV unknow |
| TCGA-29-2434 52 G3 Stage III R0 |
| TCGA-36-2552 64 G3 Stage II R0 |
| TCGA-13-0760 63 G3 Stage IV R0 |
| TCGA-24-0970 63 G3 Stage III unknow |
| TCGA-29-1776 63 G3 Stage III unknow |
| TCGA-04-1343 72 G3 Stage IV unknow |
| TCGA-25-2397 59 G3 Stage IV R0 |
| TCGA-13-2059 50 G3 Stage III R1 |
| TCGA-13-0725 44 G3 Stage III R1 |
| TCGA-09-2056 62 G3 Stage III R0 |
| TCGA-24-1464 70 G3 Stage III unknow |
| TCGA-25-1627 73 G3 Stage III R1 |
| TCGA-25-1323 72 G3 Stage III R1 |
| TCGA-61-1903 55 G3 Stage I unknow |
| TCGA-25-2042 60 G3 Stage III unknow |
| TCGA-13-0807 54 G3 Stage III unknow |
| TCGA-3P-A9WA 55 G3 Stage II unknow |
| TCGA-13-0920 65 G3 Stage III R1 |
| TCGA-29-2429 79 G3 Stage III unknow |
| TCGA-13-1500 71 G3 Stage III R2 |
| TCGA-36-2548 57 G3 Stage III unknow |
| TCGA-13-2057 59 G3 Stage III R0 |
| TCGA-42-2582 49 G3 Stage III R0 |
| TCGA-24-2029 75 G3 Stage III unknow |
| TCGA-61-2000 67 G3 Stage III R0 |
| TCGA-10-0933 77 G3 Stage III R0 |
| TCGA-13-0919 52 G3 Stage III R0 |
| TCGA-29-1778 77 G3 Stage III R0 |
| TCGA-25-1870 59 G3 Stage III R2 |
| TCGA-25-1329 76 G3 Stage III R2 |
| TCGA-13-0727 71 G3 Stage III R1 |
| TCGA-23-1024 52 G3 Stage IV R1 |
| TCGA-30-1856 56 G3 Stage III R1 |
| TCGA-61-1918 45 G3 Stage IV R0 |
| TCGA-61-2611 40 G3 Stage III R1 |
| TCGA-36-2551 57 G3 Stage III unknow |
| TCGA-24-1548 57 G3 Stage III R1 |
| TCGA-24-1927 59 G3 Stage III R1 |
| TCGA-13-1504 68 G3 Stage III unknow |
| TCGA-24-2293 47 G3 unknow unknow |
| TCGA-20-1685 45 G3 Stage III R1 |
| TCGA-29-1701 56 G3 Stage III unknow |
| TCGA-25-1626 65 G3 Stage III unknow |
| TCGA-24-1567 54 G3 Stage III R1 |
| TCGA-29-1774 82 G3 Stage III unknow |
| TCGA-29-1761 80 G3 Stage III unknow |
| TCGA-24-1428 50 G3 Stage III R1 |
| TCGA-13-0730 71 G3 Stage III unknow |
| TCGA-61-2002 46 G3 Stage III R2 |
| TCGA-09-1670 57 G3 Stage III R0 |
| TCGA-09-0367 67 G3 Stage III R1 |
| TCGA-36-2545 42 G3 Stage III R0 |
| TCGA-24-2033 87 G3 Stage III unknow |
| TCGA-10-0928 71 G3 Stage III R1 |
| TCGA-61-1727 74 G3 Stage I R0 |
| TCGA-25-1623 71 G3 Stage IV R0 |
| TCGA-24-1434 59 G3 Stage III R1 |
| TCGA-23-1031 60 G3 Stage IV R1 |
| TCGA-OY-A56Q 78 G3 Stage II R0 |
| TCGA-20-1684 51 G3 Stage III R1 |
| TCGA-24-1431 67 G3 Stage III R1 |
| TCGA-13-0901 41 G3 Stage III unknow |
| TCGA-24-1558 73 G3 Stage III R1 |
| TCGA-13-0795 66 G3 Stage III unknow |
| TCGA-10-0937 44 G3 Stage III R1 |
| TCGA-04-1517 79 G3 Stage III unknow |
| TCGA-25-2399 80 G3 Stage III unknow |
| TCGA-29-2433 61 G3 Stage III R0 |
| TCGA-59-A5PD 55 G3 Stage I unknow |
| TCGA-36-2547 64 G3 Stage III R0 |
| TCGA-25-1628 67 G3 Stage III R1 |
| TCGA-61-2109 40 G3 Stage III unknow |
| TCGA-10-0938 80 G3 Stage III unknow |
| TCGA-61-1724 47 G3 Stage III R0 |
| TCGA-09-2054 58 G3 Stage III R1 |
| TCGA-36-2544 58 G3 Stage III unknow |
| TCGA-57-1584 47 G3 Stage III R1 |
| TCGA-13-0916 49 G3 Stage III R0 |
| TCGA-13-0766 42 G3 Stage III R1 |
| TCGA-29-2432 61 G3 Stage III R2 |
| TCGA-36-1570 49 G3 Stage III unknow |
| TCGA-04-1349 69 G3 Stage IV unknow |
| TCGA-36-2542 59 G3 Stage III R1 |
| TCGA-24-0975 58 G3 Stage III R1 |
| TCGA-61-2113 53 G3 Stage II unknow |
| TCGA-24-1474 57 G3 Stage III R1 |
| TCGA-59-2350 44 G3 Stage IV R1 |
| TCGA-57-1586 66 G3 Stage III R2 |
| TCGA-24-0982 77 G3 Stage III R1 |
| TCGA-31-1951 58 G3 Stage III R0 |
| TCGA-36-1574 48 G3 Stage III unknow |
| TCGA-24-1923 51 G3 Stage III R1 |
| TCGA-36-1571 53 G3 Stage III R0 |
| TCGA-29-1769 40 G3 Stage III R1 |
| TCGA-20-0987 61 G3 Stage III R1 |
| TCGA-29-1702 84 G3 Stage III R1 |
| TCGA-25-1877 81 G3 Stage III R1 |
| TCGA-57-1582 50 G3 Stage III R1 |
| TCGA-36-1580 82 G3 Stage III unknow |
| TCGA-13-0717 54 G3 Stage III unknow |
| TCGA-36-1581 63 G3 Stage II R0 |
| TCGA-23-2072 58 G3 Stage IV unknow |
| TCGA-25-1871 70 G3 Stage III R2 |
| TCGA-36-2538 37 G3 Stage III R2 |
| TCGA-57-1993 56 G3 Stage III R0 |
| TCGA-20-1683 65 G3 Stage III R0 |
| TCGA-24-1564 67 G3 Stage III R1 |
| TCGA-36-2537 39 G3 Stage III unknow |
| TCGA-10-0926 63 G3 Stage III R2 |
| TCGA-20-0990 74 G3 Stage III unknow |
| TCGA-20-0991 78 G3 Stage II R0 |
| TCGA-23-1026 45 G3 Stage III R1 |
| TCGA-09-2055 48 G3 Stage I R0 |
| TCGA-25-1313 62 G3 Stage IV R1 |
| TCGA-24-1544 71 G3 Stage III R1 |
| TCGA-25-2409 71 G3 Stage IV R1 |
| TCGA-25-1625 66 G3 Stage III R1 |
| TCGA-13-0911 55 G3 Stage IV R1 |
| TCGA-36-1578 63 G3 Stage IV unknow |
| TCGA-61-1728 59 G3 Stage IV R1 |
| TCGA-04-1646 60 G3 Stage III R1 |
| TCGA-24-2035 65 G3 Stage III R1 |
| TCGA-24-1430 68 G3 Stage III unknow |
| TCGA-61-1734 52 G3 Stage I unknow |
| TCGA-13-0765 50 G3 Stage III R1 |
| TCGA-13-0793 40 G3 Stage IV R1 |
| TCGA-36-1568 52 G3 Stage III unknow |
| TCGA-57-1992 62 G3 Stage I R0 |
| TCGA-25-2404 38 G3 Stage III R0 |
| TCGA-36-1569 52 G3 Stage III R1 |
| TCGA-04-1536 60 G3 Stage IV R1 |
| TCGA-23-1030 64 G3 Stage III R1 |
| TCGA-09-0364 80 G3 Stage II R2 |
| TCGA-13-1483 61 G3 Stage III unknow |
| TCGA-36-2532 61 G3 Stage III R0 |
| TCGA-36-2534 72 G3 Stage III unknow |
| TCGA-13-0913 53 G3 Stage III R0 |
| TCGA-36-1576 76 G3 Stage III unknow |
| TCGA-31-1946 30 G3 Stage III R0 |
| TCGA-24-1924 65 G3 Stage III R1 |
| TCGA-13-0794 60 G3 Stage III R1 |
| TCGA-09-1669 54 G3 Stage III R0 |
| TCGA-13-0764 62 G3 Stage IV R1 |
| TCGA-61-2613 73 G3 Stage III R2 |
| TCGA-04-1365 87 G3 Stage III unknow |
| TCGA-23-1113 48 G3 Stage IV R1 |
| TCGA-29-1697 62 G3 Stage III unknow |
| TCGA-29-1768 50 G3 Stage IV unknow |
| TCGA-61-1907 63 G3 Stage III R2 |
| TCGA-61-1725 40 G3 Stage III R2 |
| TCGA-24-2271 55 G3 Stage III unknow |
| TCGA-61-1733 71 G3 Stage III R0 |
| TCGA-24-2030 87 G3 Stage III unknow |
| TCGA-23-1027 48 G3 Stage III R1 |
| TCGA-25-1325 77 G3 Stage IV unknow |
| TCGA-13-0762 65 G3 Stage III R0 |
| TCGA-04-1361 57 G3 Stage III R0 |
| TCGA-13-1501 50 G3 Stage IV unknow |
| TCGA-10-0931 44 G3 Stage III R1 |
| TCGA-36-2530 38 G3 Stage III R2 |
| TCGA-25-1314 42 G3 Stage IV R1 |
| TCGA-24-2295 69 G3 Stage III unknow |
| TCGA-23-1117 42 G3 Stage III R1 |
| TCGA-23-1123 59 G3 Stage III R2 |
| TCGA-13-1817 56 G3 Stage III unknow |
| TCGA-04-1364 61 G3 Stage III R1 |
| TCGA-61-1741 76 G3 Stage III unknow |
| TCGA-25-1321 65 G3 Stage III R0 |
| TCGA-25-1324 74 G3 Stage III R1 |
| TCGA-13-0761 51 G3 Stage IV R0 |
| TCGA-13-0912 58 G3 Stage III R0 |
| TCGA-61-1906 55 G3 Stage III R2 |
| TCGA-10-0930 70 G3 Stage III R1 |
| TCGA-59-2354 63 G3 Stage III R1 |
| TCGA-30-1861 74 G3 Stage III R1 |
| TCGA-24-2026 79 G3 Stage III unknow |
| TCGA-25-1318 54 G3 Stage III R1 |
| TCGA-36-2529 71 G3 Stage III unknow |
| TCGA-13-0804 73 G3 Stage III R1 |
| TCGA-13-0805 57 G3 Stage III R1 |
| TCGA-10-0935 68 G3 Stage III R1 |
| TCGA-09-0369 56 G3 Stage III R0 |
| TCGA-29-A5NZ 66 G3 Stage III unknow |
| TCGA-61-1738 60 G3 Stage III R1 |
| TCGA-25-1634 75 G3 Stage III R0 |
| TCGA-24-2260 74 G3 Stage III R1 |
| TCGA-04-1651 53 G3 Stage III R1 |
| TCGA-24-2290 56 G3 Stage III R1 |
| TCGA-30-1853 58 G3 Stage III R1 |
| TCGA-29-1785 55 G3 Stage III unknow |
| TCGA-29-1699 57 G3 Stage III R1 |
| TCGA-13-0792 40 G3 Stage III R0 |
| TCGA-10-0936 69 G3 Stage III unknow |
| TCGA-61-1910 56 G3 Stage II R1 |
| TCGA-13-0910 58 G3 Stage III R1 |
| TCGA-13-1498 73 G3 Stage III unknow |
| TCGA-25-1320 65 G3 Stage III R1 |
| TCGA-25-2393 81 G3 Stage III R2 |
| TCGA-30-1714 68 G3 Stage IV R1 |
| TCGA-13-1494 43 G3 Stage IV unknow |
| TCGA-25-1630 73 G3 Stage III R1 |
| TCGA-24-1616 56 G3 Stage III R1 |
| TCGA-04-1525 47 G3 Stage III R1 |
| TCGA-13-1499 56 G3 Stage III unknow |
| TCGA-09-1661 75 G3 Stage III R1 |
| TCGA-13-0791 58 G3 Stage III R0 |
| TCGA-23-1122 53 G3 Stage III R1 |
| TCGA-13-0723 63 G3 Stage III unknow |
| TCGA-OY-A56P 48 G3 Stage III R1 |
| TCGA-09-2053 72 G3 Stage III R0 |
| TCGA-61-2009 65 G3 Stage III R2 |
| TCGA-24-1557 49 G3 Stage III unknow |
| TCGA-23-1023 65 G3 Stage III R1 |
| TCGA-04-1332 70 G3 Stage III R1 |
| TCGA-24-1550 49 G3 Stage III R1 |
| TCGA-25-1326 61 G3 Stage III R1 |
| TCGA-24-1552 77 G3 Stage III R1 |
| TCGA-24-0979 53 G3 Stage IV unknow |
| TCGA-04-1516 48 G3 Stage I R0 |
| TCGA-29-1707 41 G3 Stage II R0 |
| TCGA-25-2400 76 G3 Stage III R0 |
| TCGA-25-1316 55 G3 Stage III R1 |
| TCGA-31-1955 43 G3 Stage III R0 |
| TCGA-61-1911 55 G3 Stage II unknow |
| TCGA-13-0893 48 G3 Stage III R2 |
| TCGA-61-1917 60 G3 Stage III unknow |
| TCGA-24-1435 57 G3 Stage III R1 |
| TCGA-13-0803 81 G3 Stage III R0 |
| TCGA-04-1331 78 G3 Stage III R1 |
| TCGA-31-1956 60 G3 Stage III unknow |
| TCGA-04-1362 59 G3 Stage II R1 |
| TCGA-13-0720 48 G3 Stage III R1 |
| TCGA-24-2281 68 G3 Stage II R1 |
| TCGA-61-1737 42 G3 Stage IV R1 |
| TCGA-24-2023 54 G3 Stage III R1 |
| TCGA-13-0908 58 G3 Stage IV R1 |
| TCGA-30-1860 58 G3 Stage III unknow |
| TCGA-13-0906 50 G3 Stage III R1 |
| TCGA-25-2398 71 G3 Stage III R1 |
| TCGA-29-2428 58 G3 Stage III unknow |
| TCGA-24-1466 74 G3 Stage III R1 |
| TCGA-61-2018 62 G3 Stage I R0 |
| TCGA-24-1562 67 G3 Stage III R1 |
| TCGA-31-1944 47 G3 Stage III R0 |
| TCGA-04-1338 78 G3 Stage III R1 |
| TCGA-13-0903 42 G3 Stage IV R0 |
| TCGA-24-1105 36 G3 Stage III R1 |
| TCGA-23-1021 45 G3 Stage IV unknow |
| TCGA-24-2267 58 G3 Stage II R0 |
| TCGA-24-1563 66 G3 Stage III R1 |
| TCGA-13-0905 51 G3 Stage III unknow |
| TCGA-30-1859 56 G3 unknow R1 |
| TCGA-24-1614 57 G3 Stage III R1 |
| TCGA-13-0904 63 G3 Stage III unknow |
| TCGA-04-1348 44 G3 Stage III R1 |
| TCGA-61-1736 45 G3 Stage III unknow |
| TCGA-30-1892 52 G3 Stage III R1 |
| TCGA-04-1644 48 G3 Stage III unknow |
| TCGA-61-1913 48 G3 Stage III R1 |
| TCGA-25-2391 57 G3 Stage III unknow |
| TCGA-04-1336 55 G3 Stage III R0 |
| TCGA-04-1356 62 G3 Stage II unknow |
| TCGA-23-1028 43 G3 Stage III R1 |
| TCGA-13-0894 53 G3 Stage III R1 |
| TCGA-23-1022 67 G3 Stage III R1 |
| TCGA-23-2084 45 G3 Stage IV R0 |
| TCGA-23-1109 62 G3 Stage III R1 |
| TCGA-30-1869 50 G3 Stage IV R1 |
| TCGA-61-2092 57 G3 Stage III R0 |
| TCGA-30-1718 44 G3 Stage III R1 |
| TCGA-24-1551 53 G3 Stage III R1 |
| TCGA-61-2610 61 G3 Stage III R2 |
| TCGA-25-1315 50 G3 Stage III R1 |
| TCGA-25-1635 71 G3 Stage III R1 |
| TCGA-29-1704 61 G3 Stage III R2 |
| TCGA-13-1491 55 G3 Stage III unknow |
| TCGA-24-2298 55 G3 Stage III unknow |
| TCGA-24-1103 50 G3 Stage III R1 |
| TCGA-13-1497 47 G3 Stage III unknow |
| TCGA-23-1110 42 G3 Stage III R0 |
| TCGA-13-A5FT 67 G3 Stage III R0 |
| TCGA-09-1668 57 G3 Stage III unknow |
| TCGA-04-1638 57 G3 Stage IV R0 |
| TCGA-24-2297 56 G3 Stage III unknow |
| TCGA-13-0899 60 G3 Stage III R1 |
| TCGA-24-1549 58 G3 Stage III unknow |
| TCGA-61-1914 65 G3 Stage III unknow |
| TCGA-13-0900 59 G3 Stage III unknow |
| TCGA-24-2254 66 G3 Stage III R1 |
| TCGA-24-1545 69 G3 Stage III unknow |
| TCGA-13-0897 54 G3 Stage III R1 |
| TCGA-30-1880 56 G3 Stage III R0 |
| TCGA-09-1666 57 G3 Stage III R0 |
| TCGA-09-0366 55 G3 Stage III R1 |
| TCGA-24-1553 53 G3 Stage III R1 |
| TCGA-23-1124 62 G3 Stage III R0 |
| TCGA-24-2024 72 G3 Stage III R1 |
| TCGA-25-1632 68 G3 Stage IV R1 |
| TCGA-04-1370 52 G3 Stage III R1 |
| TCGA-25-1633 64 G3 Stage III R1 |
| TCGA-29-2427 60 G3 Stage III unknow |
| TCGA-09-2051 42 G3 Stage III R0 |
| TCGA-04-1347 81 G3 Stage IV R0 |
| TCGA-24-1104 56 G3 Stage IV unknow |
| TCGA-04-1350 46 G3 Stage III unknow |
| TCGA-13-1819 59 G3 Stage II R0 |
| TCGA-24-2036 50 G3 Stage III R1 |
| TCGA-24-1546 46 G3 Stage III R1 |
| TCGA-04-1649 74 G3 Stage III R1 |
| TCGA-25-1319 73 G3 Stage III R2 |
| TCGA-25-1328 38 G3 Stage III R1 |
| TCGA-13-0887 42 G3 Stage III R1 |
| TCGA-24-2289 68 G3 Stage IV unknow |
| TCGA-61-1915 50 G3 Stage II unknow |
| TCGA-13-0890 56 G3 Stage III R1 |
| TCGA-29-1698 53 G3 Stage III R1 |
| TCGA-23-1114 55 G3 Stage III R0 |
| TCGA-13-0883 61 G3 Stage III R1 |
| TCGA-13-0891 73 G3 Stage IV R0 |
| TCGA-13-0888 78 G3 Stage III R0 |
| TCGA-24-2280 74 G3 Stage III R1 |
| TCGA-24-1556 50 G3 Stage II R1 |
| TCGA-13-1488 59 G3 Stage IV R2 |
| TCGA-04-1367 50 G3 Stage III R0 |
| TCGA-61-2094 63 G3 Stage III unknow |
| TCGA-24-1852 55 G3 Stage III R1 |
| TCGA-24-1463 70 G3 Stage III unknow |
| TCGA-13-1492 66 G3 Stage III unknow |
| TCGA-13-0886 67 G3 Stage III R1 |
| TCGA-23-2081 49 G3 Stage IV R1 |
| TCGA-59-2349 71 G3 Stage I R0 |
| TCGA-24-1930 53 G3 Stage III R1 |
| TCGA-04-1371 58 G3 Stage III R1 |
| TCGA-25-1878 60 G3 Stage III R1 |
| TCGA-23-1118 45 G3 Stage III unknow |
| TCGA-23-2078 66 G3 Stage III R0 |
| TCGA-24-1604 66 G3 Stage III unknow |
| TCGA-24-1555 50 G3 Stage III unknow |
| TCGA-09-1662 58 G3 Stage IV R1 |
| TCGA-24-1603 53 G3 Stage III unknow |
| TCGA-13-0885 70 G3 Stage III R2 |
| TCGA-23-2079 46 G3 Stage III R0 |
| TCGA-13-1484 62 G3 Stage III unknow |
| TCGA-29-1693 72 G3 Stage III unknow |
| TCGA-24-1467 51 G3 Stage III R1 |
| TCGA-13-0884 39 G3 Stage III R1 |
| TCGA-24-2027 51 G3 Stage IV unknow |
| TCGA-09-2049 64 G3 Stage III unknow |
| TCGA-23-2077 45 G3 Stage III R0 |
| TCGA-59-2351 51 G3 Stage III R1 |
| TCGA-04-1530 68 G3 Stage III R1 |
| TCGA-61-2111 61 G3 Stage IV unknow |
| TCGA-23-1119 64 G3 Stage III R1 |
| TCGA-24-2020 67 G3 Stage III R1 |
| TCGA-59-2348 59 G3 Stage III unknow |
| TCGA-24-1560 51 unknow Stage III R1 |
| TCGA-04-1353 64 unknow Stage III unknow |
| TCGA-VG-A8LO 55 unknow Stage IV unknow |
| TCGA-30-1867 46 unknow Stage IV unknow |
| TCGA-13-1410 57 unknow Stage IV unknow |
| TCGA-42-2591 51 unknow Stage III R0 |
| TCGA-5X-AA5U 61 unknow Stage II unknow |
| TCGA-13-1487 74 unknow Stage IV unknow |
| TCGA-57-1994 63 unknow unknow unknow |
| TCGA-20-1682 56 unknow Stage III R2 |
| TCGA-13-0726 55 unknow Stage III R1 |
| TCGA-29-1694 45 unknow Stage III R2 |
| TCGA-61-2110 56 unknow Stage III R0 |
| TCGA-24-2038 68 unknow Stage III R1 |
| TCGA-13-0751 44 unknow Stage III R1 |
| TCGA-13-0889 75 unknow Stage IV R1 |
